# Natural variation in a *CENTRORADIALIS* homolog contributed to cluster fruiting and early maturity in cotton

**DOI:** 10.1186/s12870-018-1518-8

**Published:** 2018-11-20

**Authors:** Dexin Liu, Zhonghua Teng, Jie Kong, Xueying Liu, Wenwen Wang, Xiao Zhang, Tengfei Zhai, Xianping Deng, Jinxia Wang, Jianyan Zeng, Yuehua Xiao, Kai Guo, Jian Zhang, Dajun Liu, Weiran Wang, Zhengsheng Zhang

**Affiliations:** 1 College of Agronomy and Biotechnology, Southwest University, Chongqing, 400716 People’s Republic of China; 2 Institute of Economic Crops, Xinjiang Academy of Agricultural Sciences, urumqi, Xinjiang 830091 People’s Republic of China; 3 Biotechnology Research Center, Southwest University, Chongqing, 400716 People’s Republic of China; 4 College of Agronomy and Biotechnology, China Agricultural University, Beijing, 100193 People’s Republic of China

**Keywords:** Cotton, Map-based cloning, Cluster fruiting, Determinate growth habit, *CEN*/*TFL1*, Plant architecture, Early maturity

## Abstract

**Background:**

Plant architecture and the vegetative-reproductive transition have major impacts on the agronomic success of crop plants, but genetic mechanisms underlying these traits in cotton (*Gossypium* spp.) have not been identified.

**Results:**

We identify four natural mutations in *GoCEN-D*_*t*_ associated with cluster fruiting (*cl*) and early maturity. The situ hybridization shows that *GhCEN* is preferentially expressed in cotton shoot apical meristems (SAM) of the main stem and axillary buds. Constitutive *GhCEN-Dt* overexpression suppresses the transition of the cotton vegetative apex to a reproductive shoot. Silencing *GoCEN* leads to early flowering and determinate growth, and in tetraploids causes the main stem to terminate in a floral bud, a novel phenotype that exemplifies co-adaptation of polyploid subgenomes and suggests new research and/or crop improvement approaches. Natural *cl* variations are enriched in cottons adapted to high latitudes with short frost-free periods, indicating that mutants of *GoCEN* have been strongly selected for early maturity.

**Conclusion:**

We show that the cotton gene *GoCEN-Dt*, a homolog of *Antirrhinum CENTRORADIALIS*, is responsible for determinate growth habit and cluster fruiting. Insight into the genetic control of branch and flower differentiation offers new approaches to develop early maturing cultivars of cotton and other crops with plant architecture appropriate for mechanical harvesting.

**Electronic supplementary material:**

The online version of this article (10.1186/s12870-018-1518-8) contains supplementary material, which is available to authorized users.

## Background

Cotton (*Gossypium* spp.) is the world’s most important natural fiber crop, a significant oilseed, and an important source of high-quality protein [1]. The *Gossypium* genus includes 45 diploid and 7 tetraploid species [2]. Two allotetraploids, *G. hirsutum* L. and *G. barbadense* (2n = 4x = 52, AADD), originating from trans-oceanic dispersal of an A-genome African species, *G. herbaceum* (A_1_) or *G. arboreum* (A_2_) (2n = 2x = 26, AA) and hybridization with an American D-genome species, *G. raimondii* (2n = 2x = 26, DD), respectively provide 95 and 2% of worldwide cotton production [1] from cultivation in about 80 countries [3].

Improved productivity to meet increased consumption of cotton fiber has heavily relied upon mechanization of traditionally labor-intensive tasks [4]. As was true in rice and maize [5, 6], genetic modification of plant architecture is of great importance to optimizing cotton for mechanized production [7]. A variety of architectures have been proposed and used to adapt to polytropic climates and to improve cotton yield potential in breeding programs [8].

Cotton has a complex growth pattern due to perennation, indeterminate growth, and sympodial fruiting of its wild ancestors [9, 10]. Indeterminate growth affects the distribution of reproductive structures, node number and yield. Determinate growth habit is conferred by a single recessive gene associated with the ‘cluster’ (*cl*) trait, of central importance for mechanical harvest [11, 12]. Now the world’s largest cotton producer, the introduction into China of the two tetraploid cultivated species in the last century included *G. barbadense* genotypes with the *cl* trait, which was incorporated into many cultivars in Xinjiang Province. Mutants of the more widely grown *G. hirsutum* L. have also been identified and used in breeding programs.

In the present study, we characterized a *cl* mutant with determinate cluster fruiting. Using map-based cloning, we identified the *GoCEN* gene corresponding to the *cl* trait. Transgenic evidence, transcriptome and polymorphism analysis show that natural mutation in *GoCEN* has facilitated cotton architecture and early maturation throughout a long history of scientific cotton breeding.

## Results

### The *cl* trait affects the distribution of reproductive structure

**Fig. 1:**
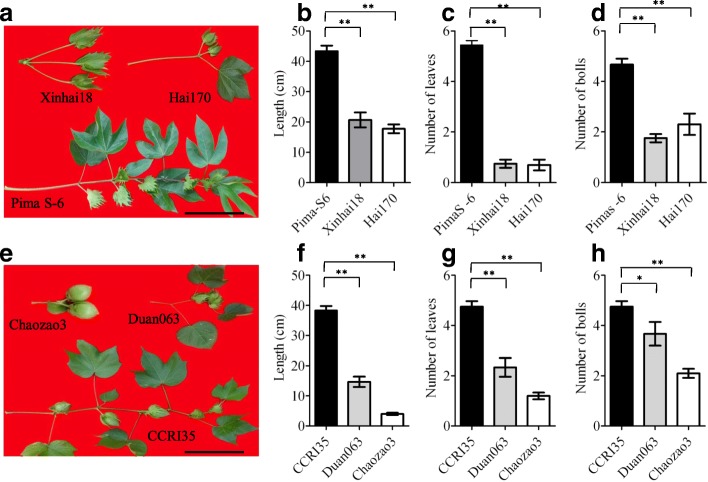
Phenotypic analyses of the first fruiting branch in wild-type and *cl-*type *G. hirsutum* and *G. barbadense*. **a**, **e** Phenotypes of the first fruit branch between reference and *cl* plants in *G. hirsutum* and *G. barbadense*. Scale bar, 10 cm. **b**, **f** Length of the first fruiting branch from stem to SAM. (**c**, **g**) Number of leaves on the first fruiting branch, including fully expanded young leaves. **d**, **h** Number of bolls and flower buds on the first fruiting branch. All data are means ± SD from 2012 to 2016 (*n* = 10 plants); * and ** represent significant differences determined by the Student’s *t*-test at *P* < 0.05 and *P* < 0.01

The *cl* trait is a crucial factor controlling plant architecture and shows wide genetic variations in *G. hirsutum* and *G. barbadense*. The wild-type growth habit is ‘indeterminate’, with continuous production of fruiting branches, and the *cl* mutant is ‘determinate’ with clustered fruit (Additional file 1: Figure S1). To quantify the impact of *cl* on cotton architecture, we compared the first fruiting branches of three Upland (*G. hirsutum* wild type CCRI35; *cl* mutants Duan063 and Chaozao3) and three Sea-Island genotypes (*G. barbadense* wild type Pima S-6; *cl* mutants Hai170 and Xinhai18). The *cl* mutants were characterized by fruiting branch termination, and reduced numbers of nodes, leaves and bolls (Fig. 1), showing that *cl* contributes substantially to variations of plant architecture and reproductive structure.

### Map-based cloning of *cl*

**Fig. 2:**
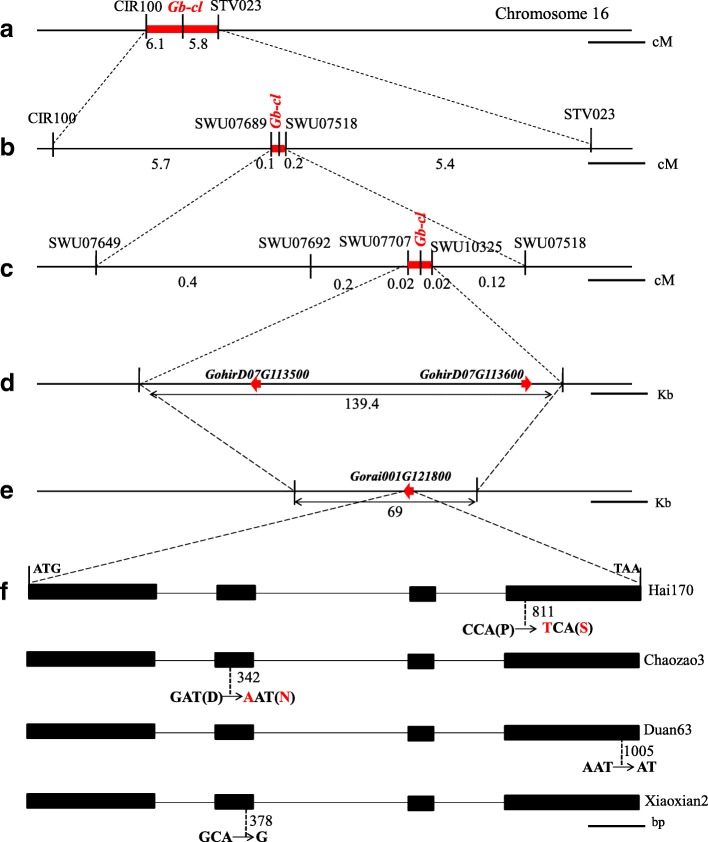
Map-based cloning of the *Gb*-*cl* gene. **a**, **b** Coarse linkage map. Scale bar, 5 cM and 1 cM in A and B, respectively. **c** High-resolution linkage map. Scale bar, 0.1 cM. **d**, **e** Delimitation to 139.4 kb and 69 kb genomic regions in *G. hirsutum* and *G. raimondii* reference genomes, each containing two or one predicted gene. Scale bar, 30 Kb. **f** Sequence diversity of *GoCEN* revealed four mutations associated with cluster fruiting in tetraploid cotton. Broken lines indicates sites of mutations. Red letters indicate non-synonymous mutations. Scale bar, 100 bp

To understand the hereditary basis of the *cl* trait, an F_2_ population including 310 progenies was constructed by crossing CCRI 35 with Hai170. The segregation ratio of normal (238) and cluster fruiting plants (72) did not deviate significantly from 3:1 (χ^2^ = 0.572 < χ^2^_0.05_ = 3.84), indicating that the *Gb-cl* trait was controlled by a single recessive gene. At least three *cl* genes are known in cotton *cl*_*1*_ and *cl*_*3*_ on chromosome 16 (D07), and *cl*_*2*_ on chromosome 7 (A07) [13]. Using 100 SSR markers on chromosome 07 and chromosome 16 from an interspecific genetic map [14], *Gb-cl* was mapped to a 600-kb region including 20 genes on chromosome 16 [9], delimited by markers CIR100 and STV023 (Fig. 2a and Additional file 1: Figure S2a). Another 559 SSR markers developed based on the *G. raimondii* genome [15] were used to screen CCRI 35 and Hai170, finding 82 markers on chromosome 16 and linked to *Gb-cl* (Additional file 1: Figure S2b and Additional file 2: Date S1). Using another 2341 F_2_ plants, *Gb-cl* co-segregated with five SSR marker was narrowed to a 0.2 cM region between markers SWU07707 and SWU08487 (Fig. 2c and Additional file 1: Figure S2c), corresponding to a 69-kb region on chromosome 01 of *G. raimondii* [15] and a 139.4 kb-region on D07 of *G. hirsutum* [16], respectively (Fig. 2d and e). Only one or two genes were annotated in this region in *G. raimondii* (*Gorai.001G121800)* or *G. hirsutum* (*GohirD07G113500* and Gohir.D07G113600)*,* and *Gorai.001G121800* and *GohirD07G113500* encoded a *TFL1-like*/*CEN-like* gene. In a second F_2_ population with 184 individuals from a cross between Duan063 and Pima-S6, *Gh-cl* was co-segregated with two SSR markers, SWU10320 and SWU7712, which also co-segregated with *Gb-cl* (Additional file 1: Figure S2c). In a third large F_2_ population with 2236 individuals from a cross between Yumian1 and Chaozao3, *Gh-cl* was narrowed to a 0.8-Mb region flanked by SWU7649 and SWU7554, containing thirty-six putative open reading frames (Additional file 1: Table S1) including *GohirD07G113500*.

Phylogenetic investigation indicated that *GohirD07G113500* is a co-ortholog of *CENTRORADIALIS* (*CEN*) in *Antirrhinum* and *SELF-PRUNING* (*SP*) in tomato (Additional file 1: Figure S4), which regulate inflorescence architecture and flowering time, respectively [11, 17]. Based on these results, we focused on *GohirD07G113500* (*GhCEN-Dt*) as a candidate for the *cl* trait.

To clarify sequence variations of *cl*, we cloned the corresponding genomic sequences from five *G. hirsutum* cultivars/lines (two wild-type, Yumian1 and CCRI 35; three *cl* mutants, Chaozao3, Duan063 and Xiaoxian2) and four *G. barbadense* cultivars/lines (two wild-type, Pima S-6 and 3–79; two *cl* mutants, Hai170 and Xinhai18). Based on the *CL* genomic sequence from *G. hirsutum* TM-1 [16], we found 3 mutations differentiating *G. hirsutum* genotypes from CCRI35 and Yumian1. In Chaozao3, an ‘A’ to ‘G’ nucleotide substitution changed amino acid Asp (D) to Asn (N); in Duan063, a single ‘A’ nucleotide acid was deleted between 1035 and 1037 bp; in Xiaoxian2, two nucleotides, ‘CA’, were deleted between 387 and 390 bp (Fig. 2f and Additional file 1: Figure S5). In *G. barbadense,* Hai170 and Xinhai18 had the same mutation: a ‘T’ to ‘C’ substitution changed proline (P) to Ser (S). Analysis of *CEN* cDNA sequences confirmed two prominent polymorphisms between *G. hirsutum* and *G. barbadense* (Additional file 1: Figure S5 and Table S2), also showing that four SNPs differentiate the At_genome and Dt_genome alloalleles.

### Spatiotemporal expression pattern of *GhCEN*

**Fig. 3:**
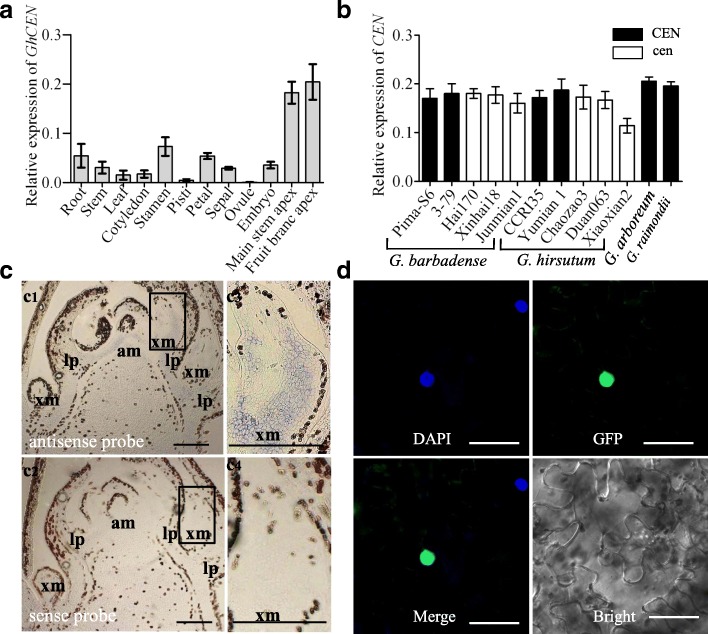
Spatiotemporal expression pattern and subcellular location of *GoCEN*. **a** Expression levels of *GhCEN* in 11 *G. hirsutum* tissues using real-time PCR. **b** Expression levels of *GoCEN* in the shoot apical meristem (SAM) of *G. barbadense* genotypes Pima S-6, 3–79, Hai 170, Xinhai18* and Junhai 1*; and *G. hirsutum* genotypes CCRI35, Yumian1, Chaozao3*, Duan063* and Xiaoxian2*; * indicates *cl* mutants. The error bars are SD of three biological replicates. **c** In situ hybridization of *GoCEN* in shoot apical meristems (SAM). Hybridization was detected with antisense probes (c1, c2) but not sense probes (c3, c4) in the SAM of Pima S-6. c2 and c4 are the enlargement of the boxes in c1 and c3, respectively. (am) apical meristem of the main stem; (lp) leaf primordium which formed leaf on the main stem; (xm) axillary meristem which formed the fruiting branch. Scale bars, 200 μm. **d** Subcellular localization of *GhCEN-GFP* in tobacco plants. Scale bars, 50 μm

Four SNPs differentiated homoeologous *GhCEN-At* and *GhCEN-Dt* transcripts (Additional file 1: Figure S5 and Table S2), but the RT-PCR primer designed to amplify *GoCEN* could not distinguish between *GhCEN-At* and *GhCEN-Dt*. Real-time PCR revealed that *GhCEN* expression was much higher in cotton main stem and fruit branch apices than in root, stem, leaf, pistil, petal, sepal, ovule or embryo (Fig. 3a). There were no significant differences in *GoCEN* expression between wild-type and mutant cottons from both *G. barbadense* and *G. hirsutum*, respectively, except Xiaoxian2 for which a two base deletion results in an open reading frame shift mutation (Fig. 2f).

The situ hybridization (Fig. 3c) revealed *GoCEN* to be expressed throughout all organ primordia. *GoCEN* is preferentially expressed in the axillary and apical meristems of the main stem, specifically in the narrow domain around the provascular bundles and in the growing tips (Fig. 3c). These results support the hypothesis that *GoCEN* plays a crucial role in the development of all axillary buds.

To determine the subcellular localization of *GhCEN*, two vectors 35S::*GhCEN-Dt-GFP* and 35S::*GhCEN-At-GFP* were transformed into *Nicotiana benthamiana*. Fluorescence detection result showed that both *GhCEN-Dt-GFP* and *GhCEN-At-GFP* were located in the nucleus of transformed tobacco plants (Fig. 3d).

### *GhCET-Dt* over-expression delays the cotton vegetative to reproductive transition

**Fig. 4:**
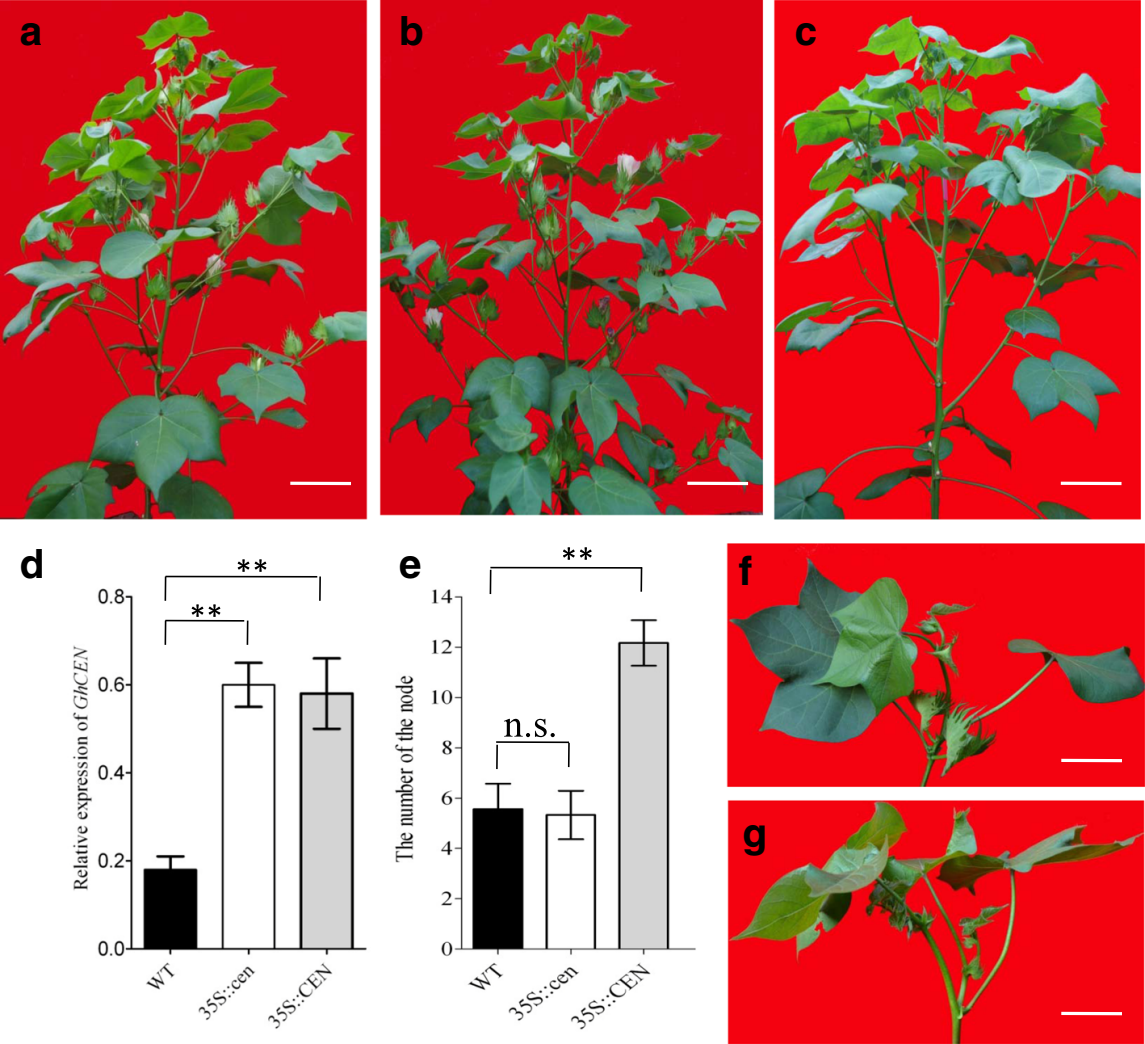
Morphologies of *GhCEN-Dt* over-expression plants. **a** Jimian 14 (wild-type) with an indeterminate phenotype. Scale bars, 15 cm. **b** Jimian 14 overexpressing *GbCEN-Dt* from Hai 170 with normal flowering time. Scale bars, 15 cm. **c** Jimian 14 overexpressing *GhCEN-Dt* from CCRI 35 with delayed flowering time. Scale bars, 15 cm. **d** Relative *GhCEN* transcript levels in transgenic lines and WT. **P < 0.01, error bars are SD of three biological replicates. **e** Position (node) of the first fruiting branch formed in WT, *35S::cen* and *35S::CEN* cottons. **P < 0.01, error bars are SD of three biological replicates; n.s., not significant. **f** and **g** Branching phenotypes of the 10th node in *35S*::*cen* (**f**) and *35S::CEN* (**g**) over-expressed cotton. Scale bars, 60 cm

To determine the function of the *GbCEN-D*_*t*_ mutation, two overexpression vectors containing full-length coding regions driven by 35S promoters, named 35S::*CEN* (from CCRI 35) and 35S::*cen* (from Hai170), were constructed and transformed into Jimian 14 (an indeterminate Upland cotton). Totals of 6 and 9 independent transgenic T_0_ lines were obtained for 35S::*CEN* and 35S::*cen*, respectively (Fig. 4a-c). Three 35S::*CEN* and three 35S::*cen* T_0_ lines with high expression were selected for further analysis (Fig. 4e). The first fruiting branch of wild-type plants usually occurs on the sixth or seventh node of the main-stem, whereas in 35S::*CEN* plants it occurred on the twelfth node (Fig. 4f). As showed in Fig. 4 g and h, at the same growth stage, *35S*::*cen* over-expressed cotton shows no difference with wild-type cotton (Fig. 4g), both having a fruiting branch on the tenth node of the main stem, but *35S*::*CEN* over-expressed cotton has a vegetative branch. This result suggests that *GhCEN* delays the vegetative to reproductive transition.

### RNAi silencing of *GoCEN* promotes cotton floral bud formation

**Fig. 5:**
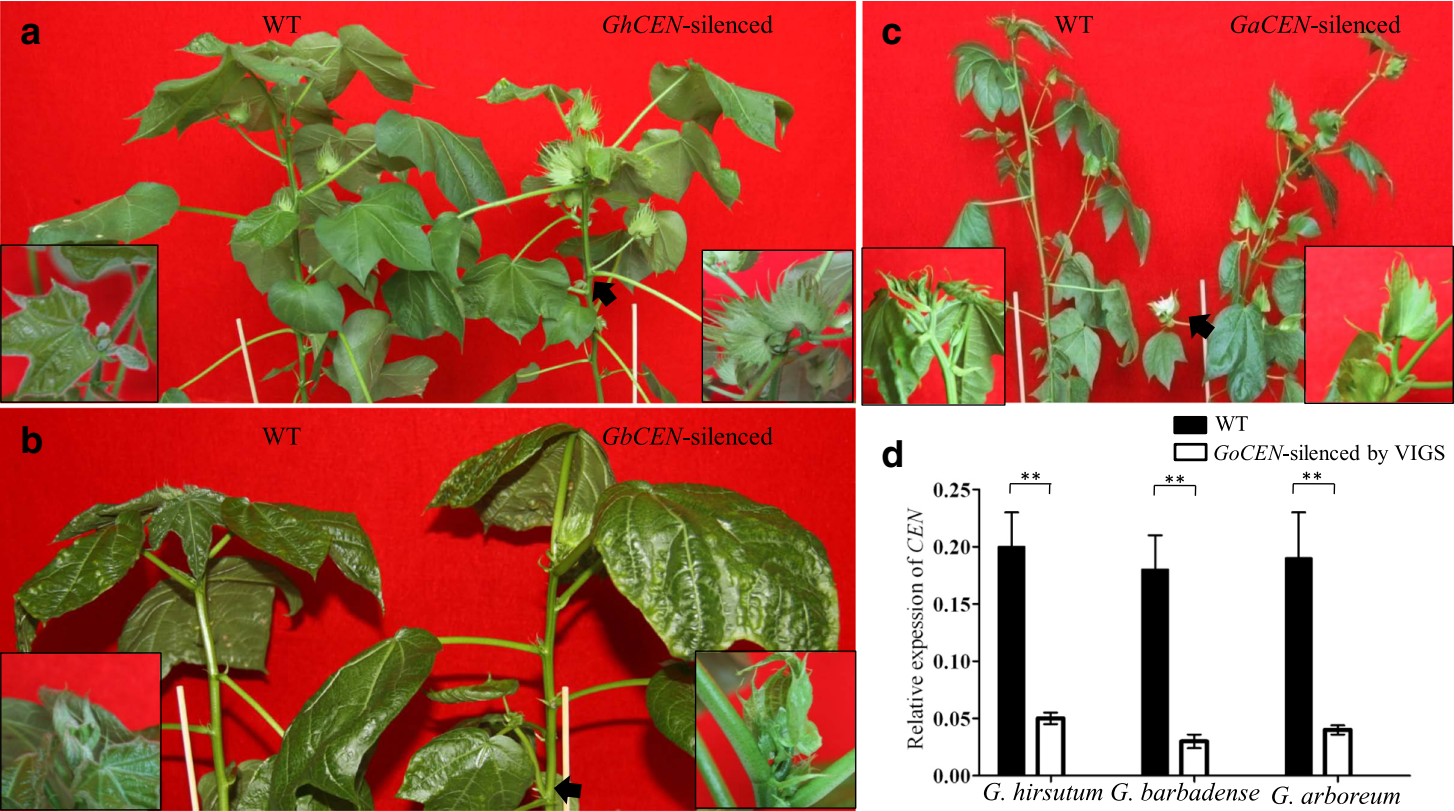
Functional characterization of *GoCEN* by virus induced gene silencing (VIGS). **a**-**c** Phenotypes of WT and *GoCEN*-silenced by VIGS in *G. hirsutum* (**a**), *G. barbadense* (**b**) and *G. arboretum* **c**; WT and *GoCEN*-silenced are on left and right, respectively. Insets are close-up views of the top of the main stem. Black arrows show the earliest floral buds in *GoCEN*-silenced cottons. **d** Relative transcript levels of *GoCEN* in WT and VIGS silenced cottons. **P < 0.01. Error bars are SD of three biological repeats

Virus induced gene silencing (VIGS) offers an attractive and quick approach to down-regulate gene expression,and VIGS vectors have been used to identify gene function in cotton [18, 19]. VIGS is environmentally sensitive and fluctuates over time [20, 21]. Therefore, a TRV:*CLA* treatment was usually used as positive control, which was sensitive and easily observable after the result of blocking chlorophyll production. In this study, numerous newly emerging photo-bleached leaves were showed in all plants inoculated with TRV:*CLA* after two weeks post inoculation (Additional file 1: Figure S6), which suggested that systemic silencing by TRV in cotton is highly potent. Then, a 241 bp fragment of *GoCEN* cDNA, corresponding to 793-1013 bp coding bases of *GohirD07G113500* from CCRI35, was cloned and inserted into the vector pTRV2. To identify the function of *GoCEN* in different *Gossypium* species, we transformed *TRV: GhCEN-silencing* fragments into *G. arboreum*, *G. barbadense* and *G. hirsutum* (Fig. 5). *GoCEN* transcript levels were significantly reduced in TRV:*GhCEN*-silenced cottons of all three species compared with the negative controls (Fig. 5d). The predicted polypeptide sequences were analyzed phylogenetically with TFL1 proteins from cotton, and two other TFL1-like clade, TFL1-L1 and TFL1-L2, shared homology with AtTFL1 [10]. We performed TR-PCR to check the change of the mRNA expression levels for TFL1-L1 and TFL1-L2, and the results showed that there is no difference between wild plants and TRV:*CEN-silenced* plants (Additional file 1: Figure S7). Floral buds emerged at the fifth node of TRV:*CEN-silenced* plants, however, no buds were observed in wild-type cotton plants in the three cultivated species. These results suggested that down-regulation of *CEN* promotes cotton floral bud formation. However, the process of the primary axis on the main stem stops the growth and the first leaf was replaced by a flower bud on the main stem in the *CEN-silenced* species (Fig. 5). This did not exist in wild-type and *cl* mutant (Fig. 1). It is well known that FT is transported from leaves to the shoot apex [22], and LFY acts as a master regulator to orchestrate the whole floral network [23]. Both FT and LFY are required for the differentiation of floral primordium. Therefore, RT-PCR was used to analyze the change of *GoFT* and *GoLYF* expression after TRV:*CEN-silenced.* The result showed that *GoLFY* significantly enhanced in TRV:*CEN-silenced* plants than wild plants, while the expression of *GoFT* in TRV:*CEN-silenced* plants was a little higher than wild plants (Additional file 1: Figure S7). The result above indicates that *GoCEN* has a fundamental role in control of the vegetative-reproductive transition and plant architecture.

### Down-regulation of *GoCEN* produces early flowering by activating MADS-box transcription factors

**Fig. 6:**
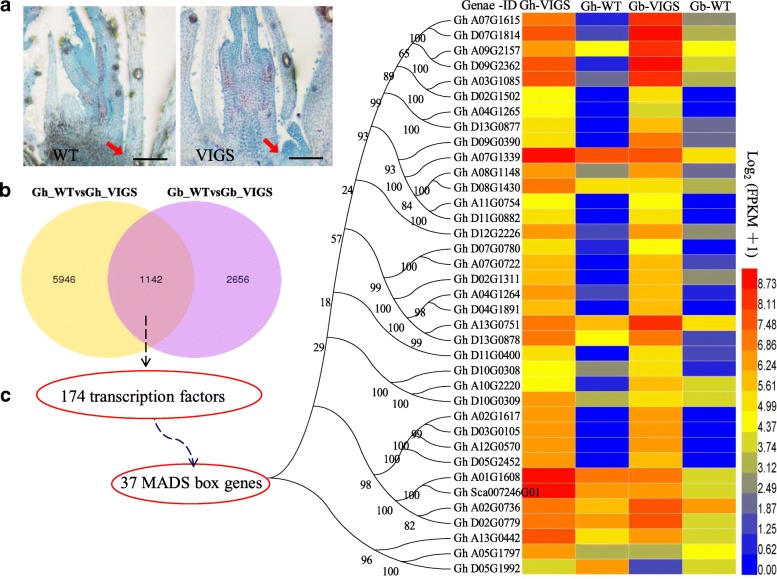
Transcriptomic comparison of SAM between WT and VIGS-silenced *GoCEN* plants in *G. hirsutum* and *G. barbadense*. **a** The SAM from wild type and VIGS-silenced *GoCEN* Pima S-6 at the three-leaf stage. Red arrows show growing points differentiated into fruit or fruiting branches. Scale bars, 100 μm. **b** Venn diagram showing the number of differentially expressed genes in WT and VIGS-silenced *GoCEN G. hirsutum* and *G. barbadense*. **c** Heatmap visualization of 37 MADS box transcription factors that are significantly differentially expressed in WT and VIGS-silenced *GoCEN* plants in *G. hirsutum* and *G. barbadense*. Red indicates up-regulated and blue indicates down-regulated expression values

Silencing *GoCEN* resulted in early production of cotton floral buds (Fig. 5a). The morphology of floral primordia was first observed in the axillary bud when the third true leaf expanded (Fig. 6a). RNA-seq data for the stem apex SAM was used to analyze transcriptional changes between *GoCEN*-silenced and WT plants, from both *G. hirsutum* (CCRI35) and *G. barbadense* (Pima S-6). A total of 1142 common genes showed differential expression between silenced *GoCEN* and WT plants in the two species (Fig. 6b and Additional file 2: Date S2). As expected, the expression level of *GoCEN* in *GoCEN*-silenced plant was lower than in WT plants (about 10 times). GO analyses revealed major enrichment of transcription factor activity (Additional file 1: Figure S8 and Additional file 2: Date S3), suggesting that transcription factors are a basis for flower formation. Further analysis revealed that 37 MADS-box transcription factors may perform important functions in cotton flower development, including 5 AP1, 6 AGL6, 3 SEP2, 2 SEP3, 2 SEP4, 3 AG, 4 AP3 and 4 PI (Additional file 1: Table S3 and Fig. 6c), which had been demonstrated to play pivotal roles in *Arabidopsis* floral induction and development [24, 25]. KEGG pathway analysis showed that these differentially expressed genes were mainly involved in secondary metabolism, plant hormone signal transduction and starch and sucrose metabolism (Additional file 1: Figure S8). Subsequent RT-PCR confirmed increased expression of some MADS-box transcription factors in *CEN*-silenced tissues (Additional file 1: Figure S9). We speculated that *CEN* protein could control the specification and differentiation of flower buds, possibly through regulating the expression of MADS box, hormone or other genes.

### Natural variation in *GbCEN* enhances early maturity

**Fig. 7:**
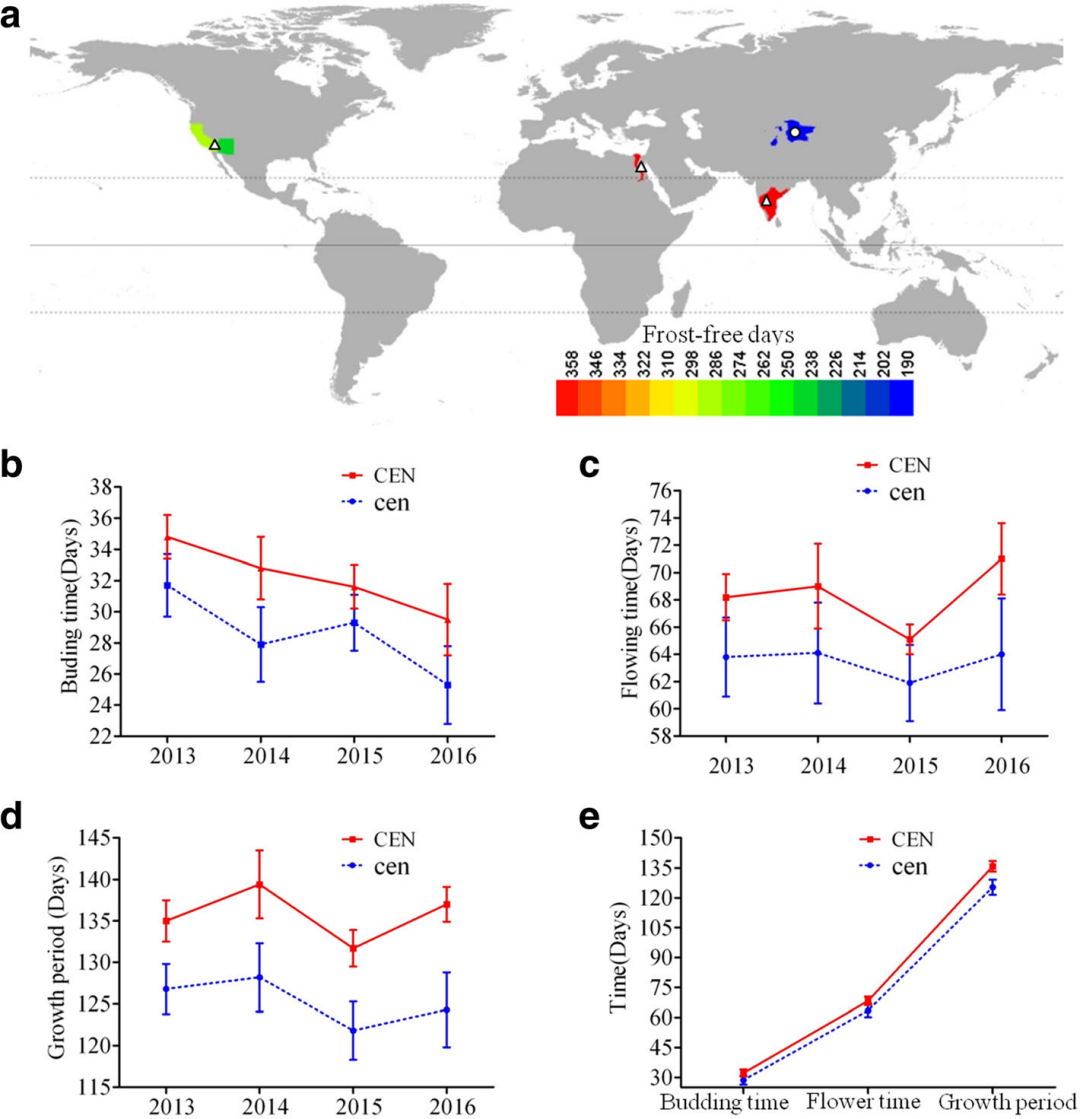
Varieties of *G. barbadense* in relation to geographic distribution and maturity. **a** Geographic distribution of the main growing areas of *G. barbadense* in the world. Colors represent the lengths of frost-free periods. Triangles and circles represent normal and *cl* fruiting branches. The world map was drawn by the authors based on the data from Resource and Environment Data Cloud Platform, DOI: 10.12078/2018110201. The data from Resource and Environment Data Cloud Platform are open and free. **b**-**e** Different types of fruiting branch of *G. barbadense* have a definite relationship with maturity. Dotted lines with corresponding colors show the average from two replicates in each year and ten mutant lines planted in a randomized complete block design. CEN and cen show the indeterminate and determinate growth habit, respectively. Error bars are SD of two biological replicates

Xinjiang Province is one of the main cotton production areas in China, and is the only region of China where *G. barbadense* is grown commercially. Compared with other cotton production regions, Xinjiang Province is located at the highest latitudes and had the shortest frost-free days (an average of 180 days per year; Fig. 7a), prompting breeders to select early flowering and maturing cultivars. Surprisingly, we found that almost all cultivars grown in Xinjiang Province are *cl* mutants (Additional file 2: Date S4), the same as the *cl* mutants, Hai170 and Xinhai18, which were further confirmed through the pedigree of those varieties (Additional file 1: Figure S10). We hypothesized that the specific natural variant *GbCEN* could accelerate the early flowering and maturing of cotton. To demonstrate this hypothesis, we collected 51 *cl* mutant lines and 41 wild type lines from the main Sea-Island cotton growing countries (Additional file 2: Date S5) and measured variation in the time of budding, flowering and maturity between the available mutants and wild types from 2013 to 2016. All *cl* mutant lines flowered and ripened earlier than wild type lines (Fig. 7b-e), indicating a significant role of *cl* variation in the adaptation of varieties to high latitudes with short frost-free periods.

## Discussion

Several independent lines of evidence support the assertion that *CEN/TFL1* genes, important regulators of plant architecture in multiple species [26], have been largely responsible for converting cultivated cottons to determinate growth habit, a trait of central importance for mechanical harvest [11, 12] that permitted cotton to become the world’s dominant textile fiber. Genetic mapping of a *cl* mutant with determinate ‘cluster fruiting’ led to identification of the *GoCEN* gene. Transgenic evidence, transcriptome and polymorphism analysis show that natural mutation in *GoCEN* has facilitated cotton architecture and early maturation throughout the history of scientific cotton breeding.

The selective advantage of determinacy associated with *GoCEN* is highlighted in that several independent mutations have each conferred determinate growth habit to different cotton species and genotypes (Fig. 2). Bradley et al. [27] and Foucher et al. [28] described similar situations wherein multiple mutants with indeterminate growth habit were caused by independent mutations in *Arabidopsis* and peas. A *G. barbadense* mutation at position 113 in the fourth exon was previously suggested to have a critical role in conferring determinacy [29]. However, to our knowledge, the mutation leading to amino acid substitution of Asp-63 with Asn in the second exon described in this study has not been previously reported in any other species. The nucleobases desertion also resulted in the reading frame shift mutation in Duan063 and Xiaoxian2.This mutation possibly inactivates the *GoCEN* protein or prevents its interaction with hypothetical cooperator(s) resulting in determinate fruiting branches with cluster fruiting.

In cotton, FD could interact with FT and CEN (TFL1 homolog), and thus antagonize FT activity in the apex and may act as transcriptional repressors of flowering transition, leading to prolonged vegetative growth [30, 31]. Though expression of FT did not significantly increase in the silencing plant, the activity of FT could further enhance interaction with FD and promoter flower. *GoCEN* may further regulate flowering time through inducing MADS-box and AUX/IAA transcription factors and activating plant hormone signal transduction. *CEN*-silencing by VIGS promoted the expression of MADS box proteins, including 8 SEP, 6 AGL6, 5 AP1, 4 PI and 3AG (Fig. 6c), that could interact with each other to regulate flowering time and floral organ formation [25]. In contrast, 6 LHY and 4 AUX/IAA transcription factor homologues are down-regulated in *GoCEN* silenced plants. LHY and SVP interact to delay flowering of *Arabidopsis* [32], and AUX/IAA transcription factors cause aberrant cotyledon placement in embryo apical patterning [33]. Sun et al. [34] also reported multi-hormone signal transduction pathways to be closely related to cotton floral induction. Uncovering the actions of these proteins will provide insight into molecular mechanisms determining plant architecture and flower formation.

Silencing CEN by VIGS conferred a novel phenotype that transgressed the *cl* mutant, with the main stem terminating in a floral bud in two tetraploid cotton species. This phenotype is consistent with a prior report [10], and may be due to the combined effects of silencing on homoeologous *GoCEN-At* and *GoCEN-Dt*. In other word, a functional *GoCEN* allele in one of the two tetraploid cotton subgenomes may maintain normal vegetative growth of the main stem apex, while silencing of both homeologs may cause the main stem to terminate in a floral bud. We mapped the *cl* trait to chromosome 16 in two tetraploid cultivated cottons, as did Chen et al. [9] and Zhu et al. [35], but Silow [36] reported that a *cl* trait from Pima cotton (*G. barbadense*) was located on homoeologous chromosome 7. In this study, We show that *GoCEN-A*_*t*_ and *GoCEN-D*_*t*_ differ by only four nucleotides and have virtually the same functionality through expression and *CEN*-silenced analysis in *G. arboreum* (Figs. 2 and 5).

Genetic dosage effects of *TFL1/CEN* in controlling fruiting type and flowering time in allotetraploid cotton have important implications. *TFL1/CEN* had been researched in diploid species by positional cloning [10]. However, our discovery of non-additive consequences of silencing multiple, functionally-similar gene family members provide an intriguing example of how polyploid subgenomes have co-evolved following their merger in a common nucleus. Moreover, this finding implies that discovery or engineering of genotypes with additional *TFL1/CEN* copies (for example, by natural single gene duplication, or artificial genome editing) could result in novel phenotypes, for example conferring extreme determinacy or rapid flowering. This implication suggests new approaches to research on plant architecture and flower formation, as well as new opportunities for seeking or creating novel crop phenotypes using *CEN* genes.

The close association of *cl* variation with adaptation of varieties to high latitudes with short frost-free periods suggests opportunities to extend the productive range of cotton and other crops. *TFL1/CEN* had been reported to play crucial roles in maintaining shoot formation by SAM [11, 27]. In the present study, *GhCEN* is shown to be highly expressed in the SAM of the main stem and axillary buds, with overexpression of *CEN-Dt* postponing the floral transition while *CEN*-silencing advanced flowering in cotton. Thus, *cl* can accelerate flowering, providing a probable explanation for its abundance in *G. barbadense* cottons bred for high latitudes and low density early planting. Likewise, introduction of the recessive *sp* gene into tomato cultivars resulted in determinate growth habit and facilitated mechanical harvesting [11], and bean and pea also have determinate varieties [37–39]. Therefore, the suppression of CEN may provide a strategy for improvement of plant architecture and better adaptation of cotton and other crops to cultivation in regions with short frost-free periods.

## Conclusions

In the present study, using a map-based cloning strategy, we have successfully cloned a gene *GoCEN-D*_*t*_, a homolog of Antirrhinum *CENTRORADIALIS*, which is responsible for the four natural mutations in the determinate growth habit with cluster fruiting (*cl*) in cotton. The overexpression of *GhCEN-Dt* suppresses the transition of the vegetative apex to a reproductive shoot, whereas silencing *GoCEN* leads to early flowering and determinate growth habit in all apices. We evaluated the importance of the CEN gene for plant architecture and flowering transition in cotton. The *cl* mutation as marker trait has been selected in cotton breeding in China because of the mutation related with early-maturity. The mutation in CEN gene can increase global cotton production and accelerate flowering, providing a probable explanation for its abundance in cotton bred for high latitudes and high density early planting.

## Methods

### Plant materials and growth conditions

Two *G. barbadense* cluster fruiting mutants, Xinhai18 and Junhian1 of and two *G. hirsutum* cluster fruiting mutants Chaozao3 and Xiaoxian2, were provided by the National Medium-term Gene Bank of Cotton in China and National cotton germplasm resources platform. Cluster fruiting mutant Hai170 was provided by the Agricultural Sciences Institute for the seventh divisions of Xinjiang Production and Construction Corps. *Gossypium hirsutum* cultivar CCRI35 and *G. barbadense* line Hai170 were chosen to produce the segregating population. CCRI 35, a normal fruiting type, was widely planted in China in the last decade. The population was developed in summer 2011 at Southwest University (SWU), Chongqing, China. Individual F_1_ plants were self-pollinated and F_2_ seeds were harvested in winter 2011 in Sanya, Hainan, China. Parents and F_2_ segregating population were planted in single-row plots 0.7 m wide and 5 m long, in 2012 and 2013 at Southwest University (SWU), Chongqing, China.

An additional 92 accessions collected from the main Sea Island cotton growing countries, including 51 *cl* mutant lines and 41 normal type lines, were grown in Korla, Xinjiang Province, China from 2013 to 2016 and were used to investigate the relationship between the type of fruiting branch and maturity (Additional file 2: Data S5).

### Map-based cloning of *GoCEN*

Genomic DNA from parents and the segregating population was isolated by CTAB method. Simple sequence repeat (SSR) primers with prefix SWU are described in Additional file 2: Data S1. All primers were synthesized by Invitrogen (Shanghai, China). Genotyping using SSR markers was done as described [40]. Only the clear DNA bands on the gels were chosen for scoring and genotypes were scored according to the coding system described in the JoinMap4.0 Manual.

Based on the *G. raimondii* genome [15], *Gorai.001G121800* was amplified with primers encompassing the coding regions. The 25-μl PCR reactions included 50 ng cotton genomic DNA or cDNA, 1 × PrimerSTAR mix (TaKaRa), and 200 nM upstream and downstream primers. The primers were listed in Additional file 2: Data S5. *GoCEN* genomic sequences were amplified from two diploid (*G. arboreum*, *G. raimondii*) and two tetraploid species (*G. hirsutum, G. barbadense*). The PCR thermal cycling parameters were: 98 °C for 1 min, followed by 35 cycles of 98 °C for 10s, 55 °C for 15 s and 72 °C for 30s, and a final extension of 5 min at 72 °C. PCR products were cloned into PDM19-T vector (TaKaRa) and sequenced by Invitrogen (Shanghai, China). In tetraploid cotton, *GoCEN* has two homeologs (one similar to the diploid D genome and the other to the A genome), named *GoCEN-Dt* and *GoCEN-At*, respectively.

### Plasmid construction and plant transformation

To construct overexpression vectors, fragments of the full length *CEN-Dt* from CCRI35 and Hai170 were cut from PDM19-T with *EcoRI* and *SmaI* restriction enzymes, gel purified, and cloned into pPLGN-35S-MCS-Nos to produce the constitutive overexpression constructs.

Overexpression constructs were transformed into *Agrobacterium tumefaciens* (LBA4404), and the resulting strains were used to produce transgenic cottons. Jimian 14 cotyledons were used as explants for transformations according to the protocol of Luo et al. [41] Transgenic plantlets were identified using histochemical GUS staining in leaf tissues, and GUS-positive plants were transplanted and grown in the greenhouse. PCR was used to confirm the presence of transgenes in transformants and progeny of transgenic lines.

### VIGS of *GoCEN* in cotton

A *GhCEN* cDNA fragment was cloned from the SAM of Upland cotton CCRI35 vegetative shoots. A 241-bp fragment from the fourth exon was amplified from cDNA. The primers were listed in Additional file 2: Data S1. The product was cloned into PDM19-T vector (TaKaRa) and sequenced by Invitrogen (Shanghai, China). *EcoRI* and *KpnI* inserts were cloned into TRV2, generating a TRV2-GoCEN vector. Binary vectors (TRV1 and TRV2-GhCEN) were introduced into *Agrobacterium tumefasciens* strain GV3101 by electroporation. Three cultivated species, *G. arboreum*, *G. barbadense* and *G. hirsutum*, were infiltrated with a 1:1 mixture of *Agrobacterium* carrying pTRV1 and TRV2 as a negative control, or pTRV1 and TRV2-GOCEN at 1 week post-germination using a 1-ml syringe. The VIGS assay was used to inoculate cultures as described [42]. Inoculated plants were first kept for 48 h (hour) at room temperature in darkness, then transplanted into a growth chamber under a 26/22 °C day/night cycle in long-day conditions (16 h/8 h, light/dark).

### RNA sample extraction and real-time PCR analysis

Total RNAs were extracted from roots, stems, leaves, petals, ovules and SAM, using a rapid plant RNA extraction kit (Aidlab, Beijing, China). The cDNAs were synthesized from total RNA using a first-strand cDNA synthesis kit (TaKaRa, Dalian, China), and then subjected to real-time PCR analyses. Real-time PCRs were performed on a CFX96 real-time PCR detection system using SYBR Green Supermix (Bio-Rad, CA, USA) according to the manufacturer’s introductions. The thermal cycling parameters were: 95 °C for 2 min, followed by 40 cycles of 95 °C for 10 s, 57 °C for 20 s, followed by a standard melting curve to monitor PCR specificity. The primers are listed in Additional file 2: Data S1. Data were analyzed using the software Bio-Rad CFX Manager 2.0 provided by the manufacturer.

### In situ hybridization

A 221-bp gene-specific *CEN* probe was amplified with the primers Prob-CEN-F and Prob-CEN-R (Additional file 1: Data S1) and labeled using the DIG RNA Labeling Kit (SP6/T7; Roche), following the manufacturer’s recommendations. Pretreatment of sections, hybridization and immunological detection were performed using published methods [43]. Source tissue was obtained from the tips of main stems, including embryonic leaves, apex tissues and non-elongated internodes at the full-bloom stage.

### Subcellular localization

The coding regions of *GoCEN-At* and *GoCEN-Dt* without the stop codon were amplified from CCRI35 cDNA (*G. hirsutum*) using primers (Additional file 2: Data S1). The following constructs were obtained and confirmed by sequencing: *35S::GhCEN-At-GFP* and *35S::GhCEN-Dt-GFP*. Both constructs were introduced into *N. benthamiana* plants by *Agrobacterium tumefasciens* GV3101. After 48 h incubation, GFP fluorescence and DAPI was observed and co-localized by confocal laser scanning microscopy (FV1000; Olympus, Tokyo, Japan).

### Transcriptome analysis

Shoot apical meristem (SAM) tissue was used for RNA-seq to identify DEGs in *CEN* silenced *G. hirsutum* and *G. barbadense*. Flower bud differentiation is first observed at the expansion of the third true leaf using VIGS of *CEN* in *G. hirsutum* and *G. barbadense* (Fig. 6a). After a period of growth in chamber, SAMs without leaves from *G. hirsutum* CCRI35, *CEN* silenced CCRI35 and *G. barbadense* Pima S-6, *CEN* silenced Pima S-6 were collected for total RNA extraction. Two biological replicates were performed. RNA extraction, detection and library construction followed our published method [40]. Library preparations were sequenced on an Illumina Hiseq platform by Novogene Bioinformatics Institute (Beijing, China) and 125 bp/150 bp paired-end reads were generated. The clean reads were obtained by removing reads containing adapter, reads containing ploy-N and low quality reads from raw data. The clean sequence tags were mapped to a *G. hirsutum* (TM-1) genome [16] using TopHat v2.0.12 [44]. FPKM [45] (Fragments Per Kilo base of exon per Million fragments mapped) was used to screen differentially expressed genes (DEGs) between pairwise comparisons (*CEN*-silenced CCRI35 versus CCRI35 and *CEN*-silenced Pima S-6 versus Pima S-6). Differentially expressed genes (FDR adjusted *P* value ≤0.05 and log2 fold change ≥1) were identified by performing a pair wise comparison. Gene Ontology (GO) terms with corrected P value less than 0.05 were considered significantly enriched using GOseq R package [46]. Pathway analysis was mainly based on the Kyoto Encyclopedia of Genes and Genomes (KEGG) database [47].

## Additional files

Additional file 1: **Figure S1.** Plant morphologies of WT and mutants with *cl* trait in *G. barbadense* and *G. hirsutum*. **Figure S2.** Fine mapping of *Gb*-*cl*. **Figure S3** Gnentic mapping of *Gh*-*cl*. **Figure S4.** Phylogenetic tree of TFL1-related proteins constructed using neighbor-joining method with the program MEGA 5.10 in tree view. **Figure S5.** The nucleotide sequence alignment of *GoCEN* genesfrom A-subgenome and D-subgenome from cotton cultivars/lines used in this study. **Figure S6.** Functional characterization of Go*CLA* by VIGS. **Figure S7.** Genes expression level with RT-PCR in *G. hirsutum* and *G. barbadense* between CEN-silenced and WT plant. **Figure S8.** GO and KEGG enrichment analysis of differentially expressed genes screening from WT and *GoCEN* silenced plant in *G. hirsutum* and *G. barbadense*. **Figure S9.** qRT-PCR validation of MADS-box transcription factors in *G. hirsutum* and *G. barbadense* between CEN-silenced and WT plant. **Figure S10.** The pedigree of *G. barbadense* commercial cultivars in Xinjiang Province. **Table S1.** Thirty-six candidate genes for *Gh-cl* and their putative function. **Table S2.** Nucleotide sequence variations between *GoCEN-A07* and *GoCEN-D07* in tetraploid cotton. **Table S3.** MADS-box transcription factors differentially expressed in transcriptome. (DOCX 5482 kb)

Additional file 2: **Data S1.** All primers used in this study. **Data S2.** The list of significant differentially expressed genes in Gh_VIGSvsGh_WT_Gb_VIGSvsGb_WT.venn. **Data S3.** 174 transcription factors in Gh_VIGSvsGh_WT_Gb_VIGSvsGb_WT.venn. **Data S4.** The 58 cultivars bred in Xinjiang Province. **Data S5.** The information of the expermental material of *G. barbadense*. (XLSX 167 kb)

## Abbreviations

*CEN*: *CENTRORADIALIS*
CL: Cluster fruiting
DEGs: Differentially expressed genes
GFP: Green fluorescent protein
GO: Gene Ontology
KEGG: Kyoto Encyclopedia of Genes and Genomes
qRT-PCR: Quantitative real-time PCR
RNA-seq: RNA sequencing
SAM: Shoot apical meristems
SSR: Simple sequence repeat
VIGS: Virus induced gene silencing
WT: Wild-type
