# Supplementary material for: Natural variation in a CENTRORADIALIS homolog contributed to cluster fruiting and early maturity in cotton
Source: BMC Plant Biol. 2018 Nov 20;18:286. doi: 10.1186/s12870-018-1518-8 (PMC6245773; doi:10.1186/s12870-018-1518-8)
Supplement: Supplementary file 1 — Figure S1. Plant morphologies of WT and mutants with cl trait in G. barbadense and G. hirsutum. Figure S2. Fine mapping of Gb-cl. Figure S3 Gnentic mapping of Gh-cl. Figure S4. Phylogenetic tree of TFL1-related proteins constructed using neighbor-joining method with the program MEGA 5.10 in tree view. Figure S5. The nucleotide sequence alignment of GoCEN genesfrom A-subgenome and D-subgenome from cotton cultivars/lines used in this study. Figure S6. Functional characterization of GoCLA by VIGS. Figure S7. Genes expression level with RT-PCR in G. hirsutum and G. barbadense between CEN-silenced and WT plant. Figure S8. GO and KEGG enrichment analysis of differentially expressed genes screening from WT and GoCEN silenced plant in G. hirsutum and G. barbadense. Figure S9. qRT-PCR validation of MADS-box transcription factors in G. hirsutum and G. barbadense between CEN-silenced and WT plant. Figure S10. The pedigree of G. barbadense commercial cultivars in Xinjiang Province. Table S1. Thirty-six candidate genes for Gh-cl and their putative function. Table S2. Nucleotide sequence variations between GoCEN-A07 and GoCEN-D07 in tetraploid cotton. Table S3. MADS-box transcription factors differentially expressed in transcriptome. (DOCX 5482 kb) [file 12870_2018_1518_MOESM1_ESM.docx]

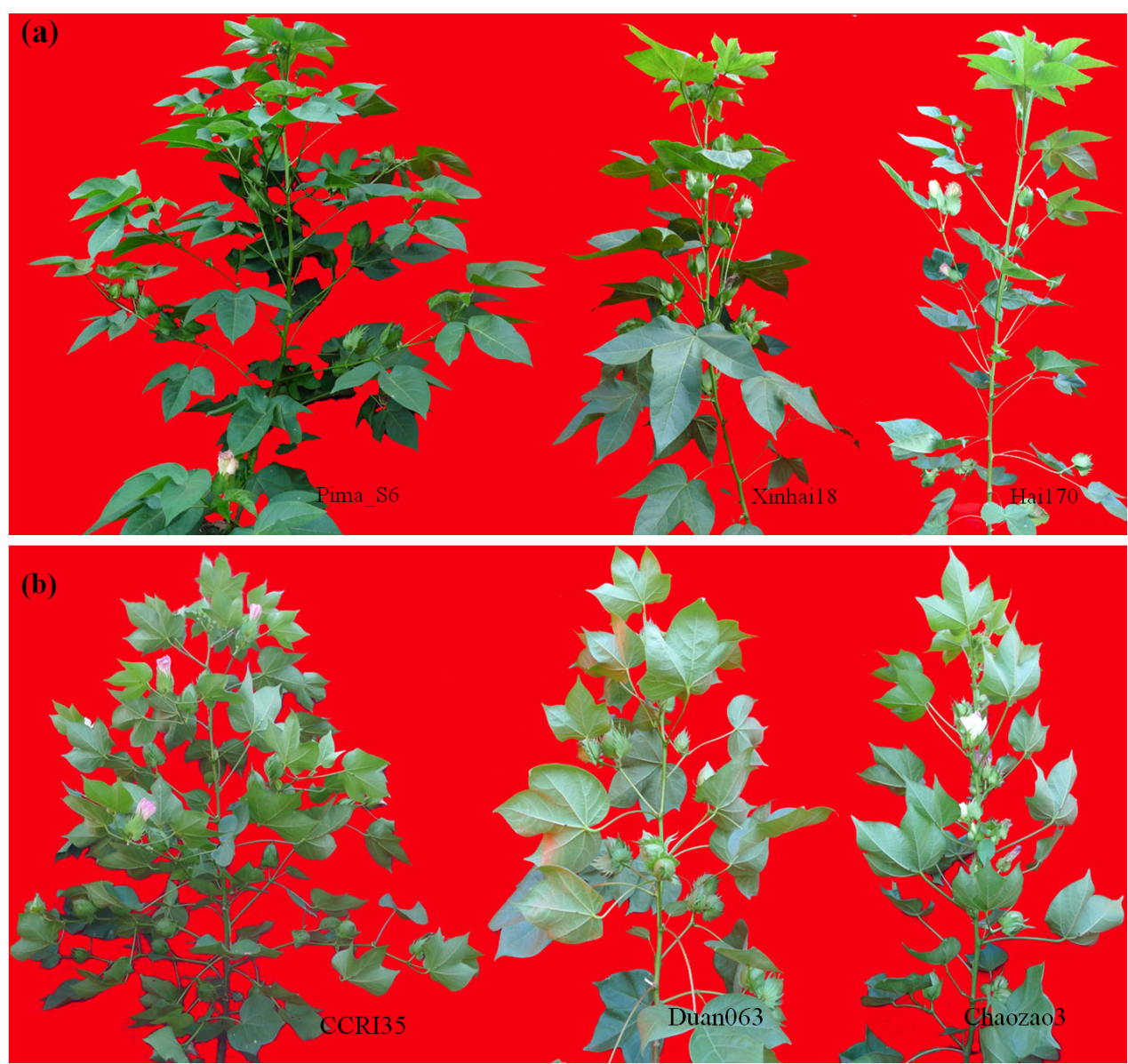


**Fig. S1. Plant morphologies of WT and mutants with *cl* trait in *G. barbadense* and *G. hirsutum***. (a) Plant architecture in *G. barbadense*. Pima-S6 (WT) with indeterminate growth habit; Xinhai18 and Hai170 (mutant) in determinate growth habit with clustering fruit. (b) Plant architecture in *G. hirsutum*. CCRI35 (WT) with indeterminate growth habit; Duan063 and Chaozao3 (mutant) in determinate growth habit with clustering fruit. Scale bar, 20 cm.


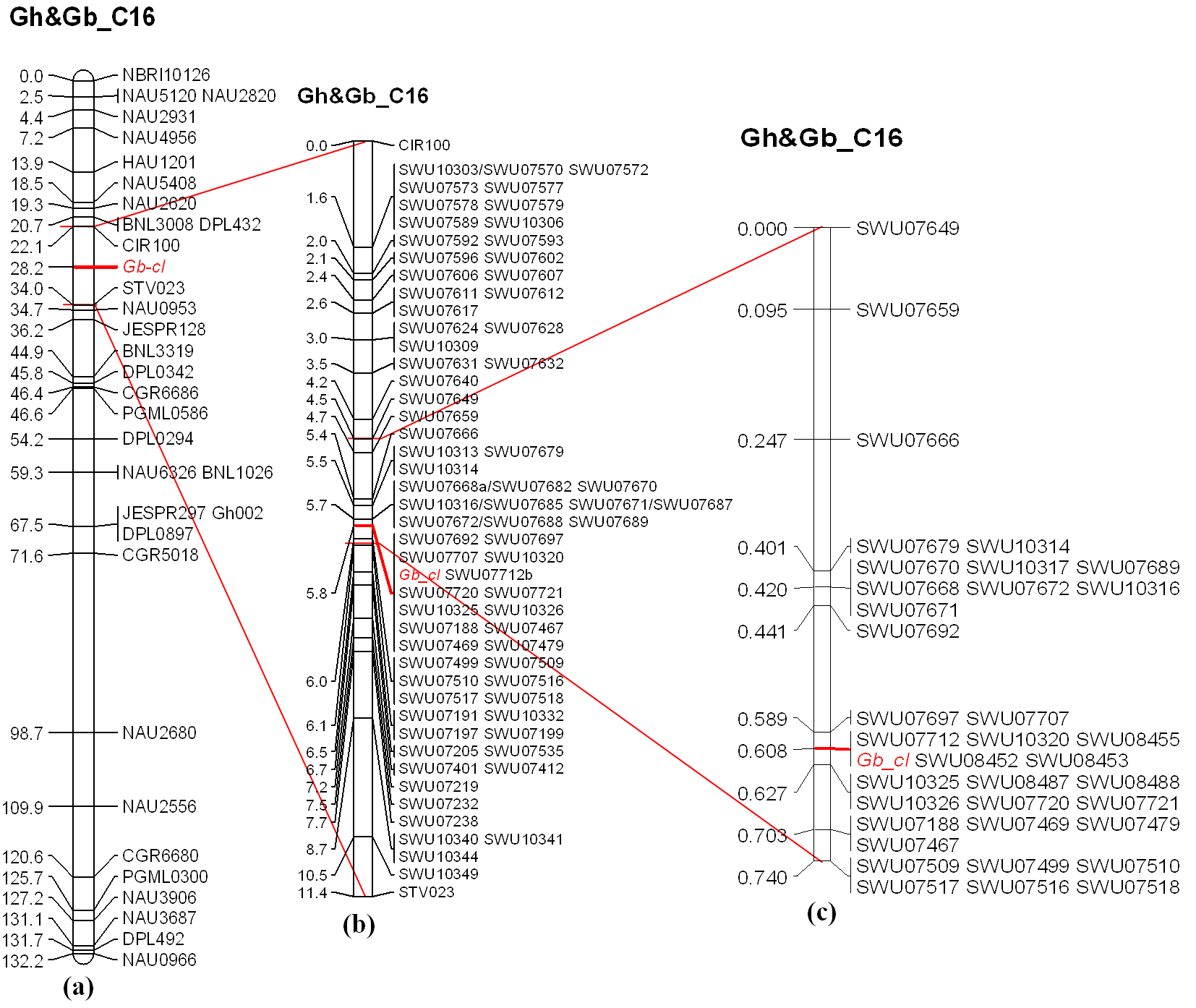


**Fig. S2.** **Fine** **mapping of *Gb*-*cl***. (a) Preliminary location of *Gb*-*cl*. *Gb*-*cl* was mapped on chromosome 16 with 90 plants from (*G. hirsutum* cultivar CCRI 35 × *G. barbadense* line Hai170) F_2_ population. (b) The high-density genetic map of *Gb*-*cl* region. *Gb*-*cl* was further mapped to an interval flanked by SWU07689 and SWU 07518 and co-segregated with 13 markers. New SSR primer was showed in Supplementary Data 1. (c) Fine mapping *Gb*-*cl* using a large F_2_ population with 2341 plants. *Gb*-*cl* was mapped to a 0.019-cM region between marker SWU07707 and SWU08487 and co-segregated with 5 markers.


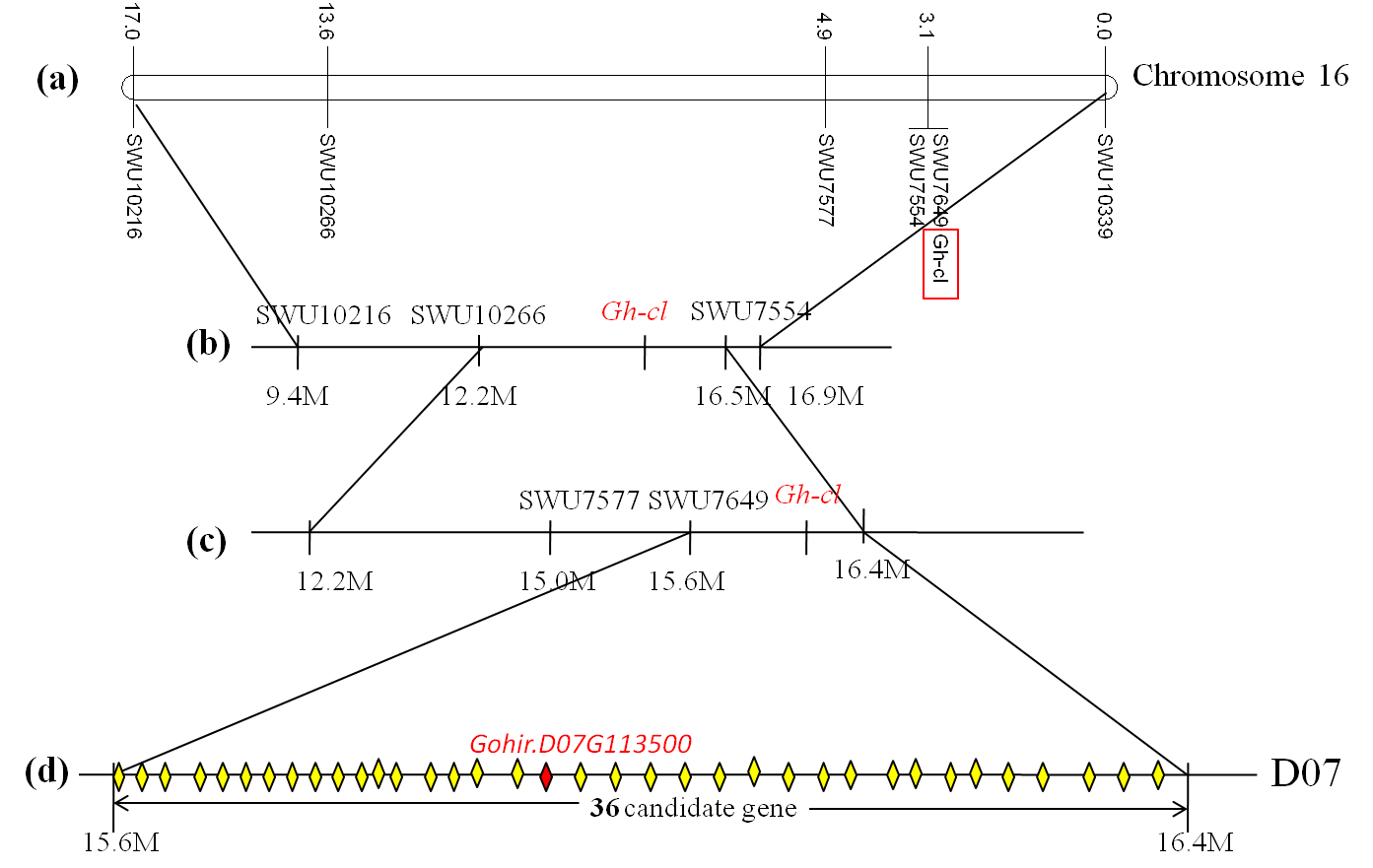


**Fig. S3.** **Gnentic mapping of *Gh*-*cl***. (a) The preliminary location of *Gh*-*cl*. *Gh*-*cl* was maped on chromosome 16 using a *G. hirsutum* intraspecific F_2_ population with 90 plants from a cross between Yumian1 and Chaozao3. (b and c) Fine mapping of *Gh*-*cl*. *Gh-cl* was narrowed to a 0.8-Mb region ﬂanked between SWU7649 and SWU7554 with a large F_2_ population with 2236 plants. (d) Physical mapping for *Gh*-*cl*. Thirty-six putative open reading frames including *Gohir.D07G113500* are predicted within this region, based on *G.hirsutum* acc.TM-1 (see Additional file 2: Date S1).


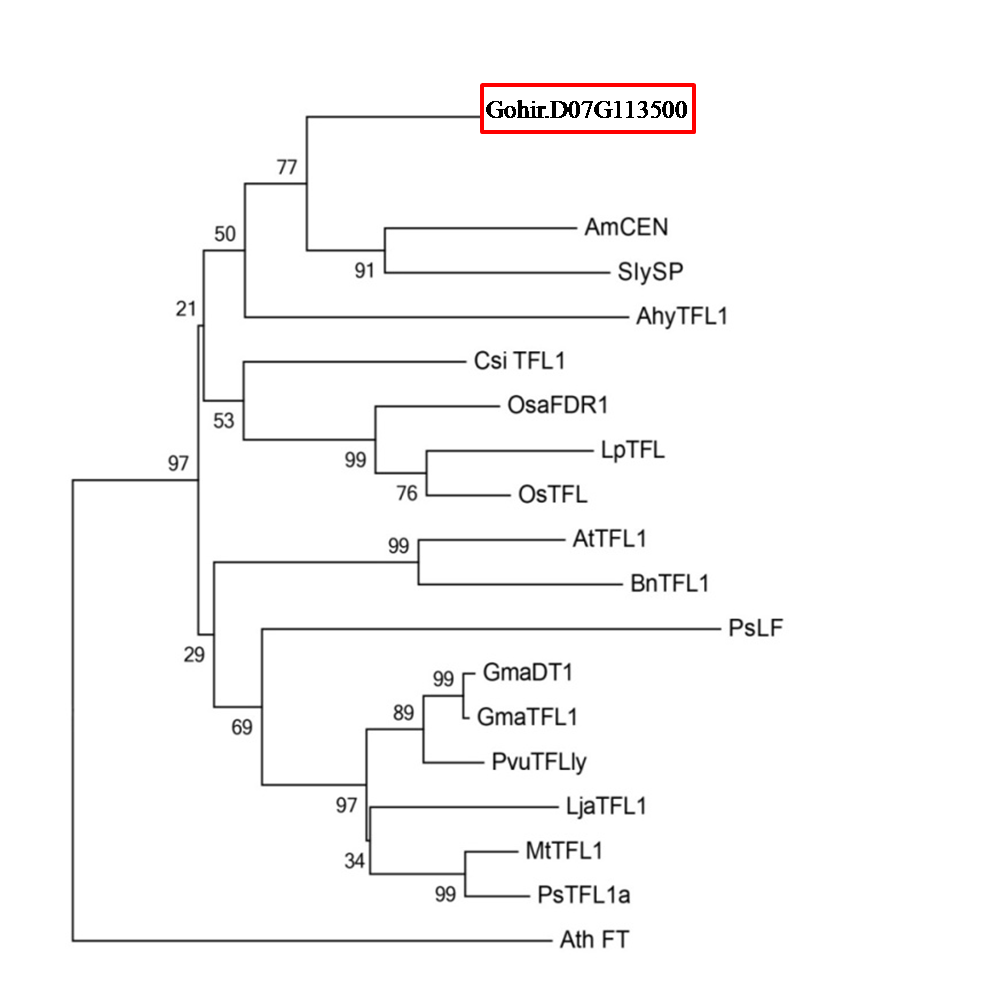


**Fig. S4.** **Phylogenetic tree of TFL1-related proteins constructed using neighbor-joining method with the program MEGA 5.10 in tree view.** The accession numbers of the known proteins of sequences in NCBI are as follows: *Antirrhinum majus*-AmCEN (AAB36112.1); *Solanum lycopersicum*-SlySP (NP_001233974); *Arachis hypogaea*-AhyTFL1(AFP33421); *Citrus sinensis*-CsiTFL1 (NP_001275848); *Oryza sativa*-OsaFDR1 (AAD42896); *Lolium perenne*_LpTFL(AAG31808); *Oryza sativa*-OsaFDR1 (AAD42896); *Oryza sativa*_OsTFL (AAD42895); *Arabidopsis thaliana*-AthTFL1 (NP_196004); *Brassica napus*-BnTFL1 (BAA33415); *Pisum sativum*-PsTFL1 (AAR03725); *Pisum sativum*-PsLF (AAQ20811); *Glycine max*-GmaDT1 (ADF30943); *Glycine max*-GmaTFL (ACU00123); *Phaseolus vulgaris*-PvuTFLly (ABR53775); *Lotus japonicus*-LjaTFL1 (AAQ93599); *Medicago truncatula*-MtTFL1 (XP_003625808); *Arabidopsis thaliana*-AthFT (BAA77838).


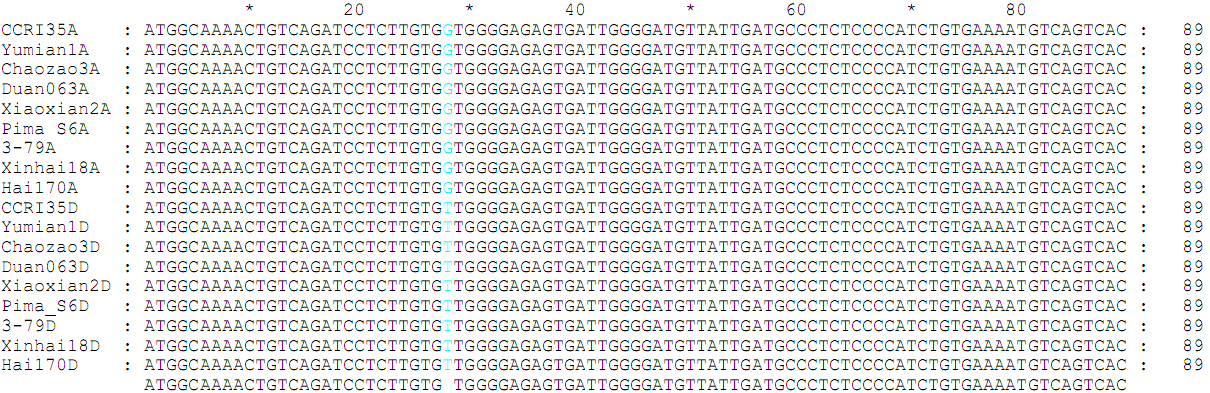


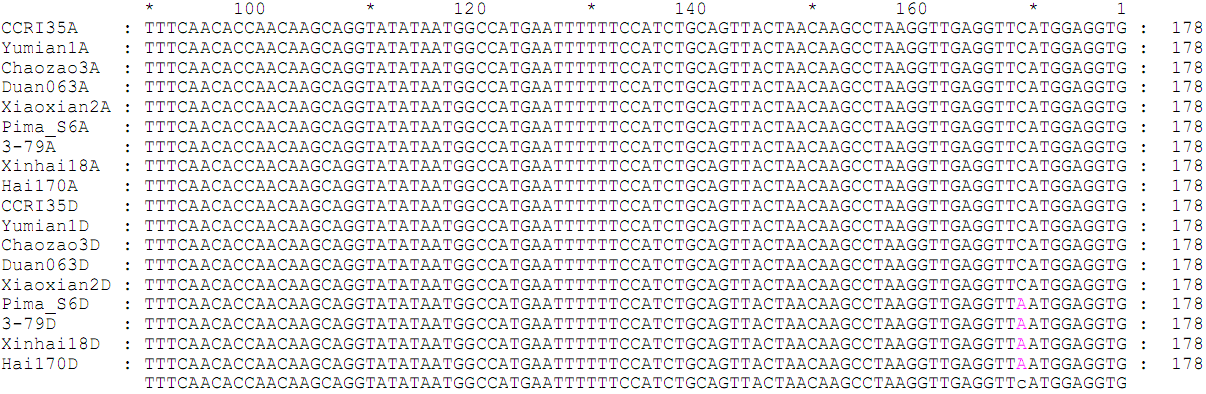


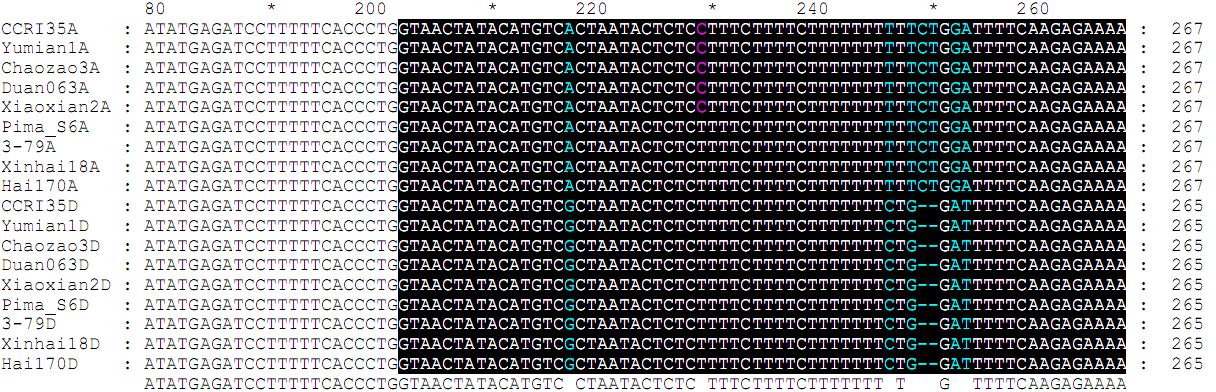


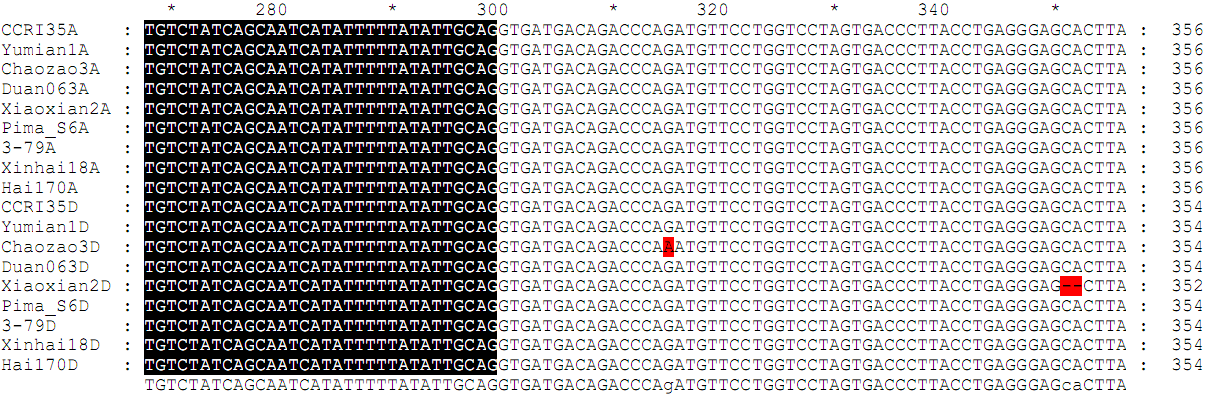


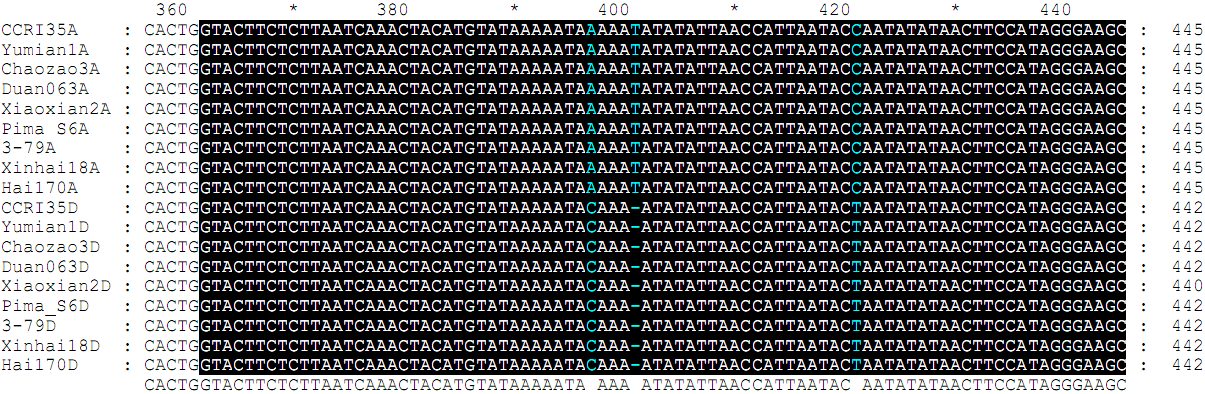


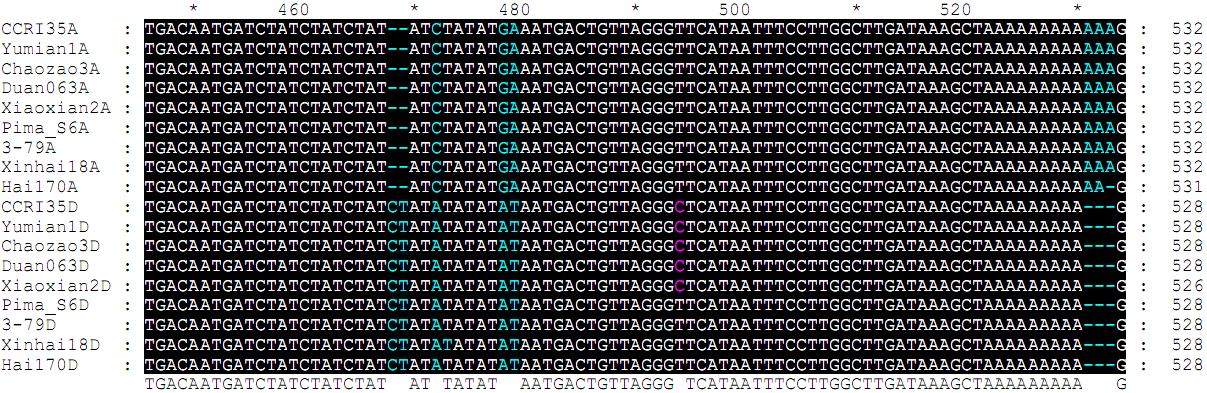


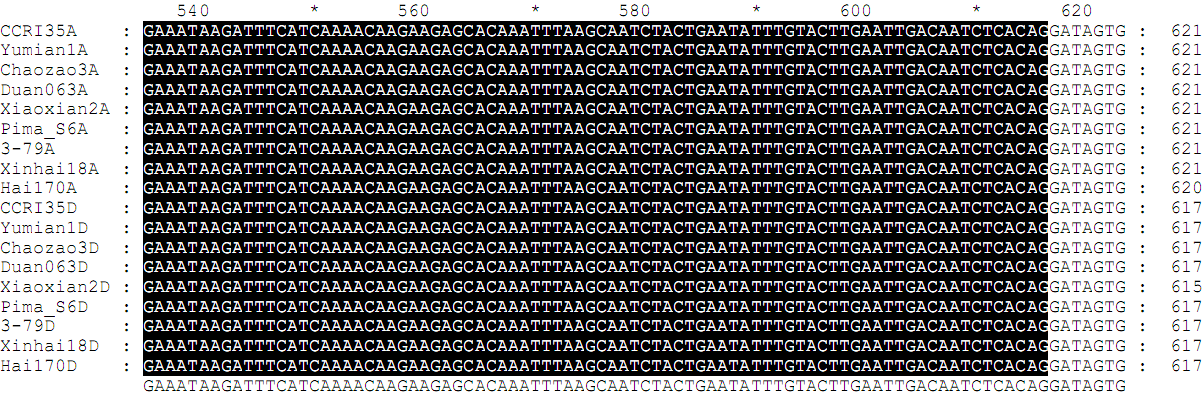


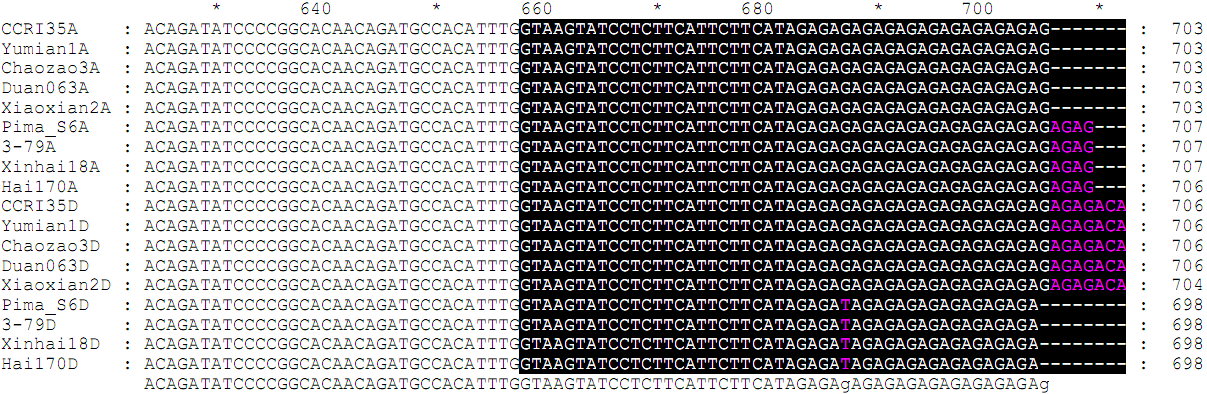


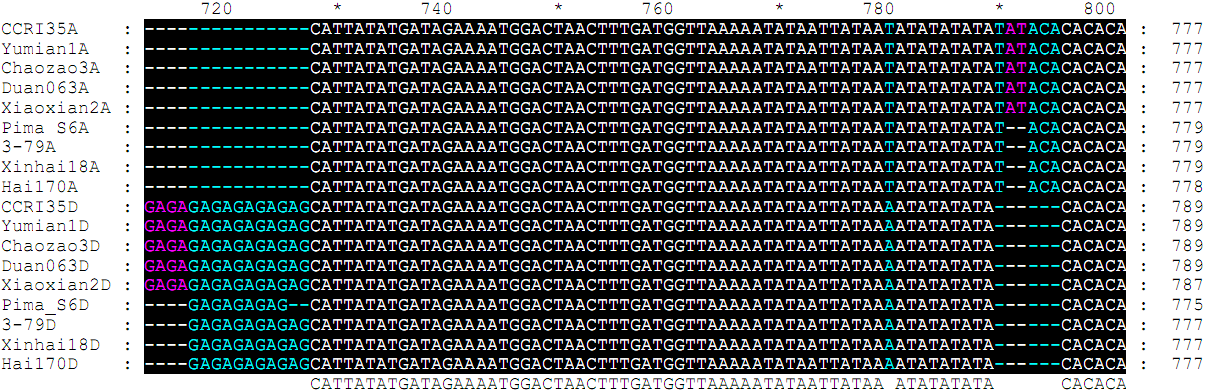


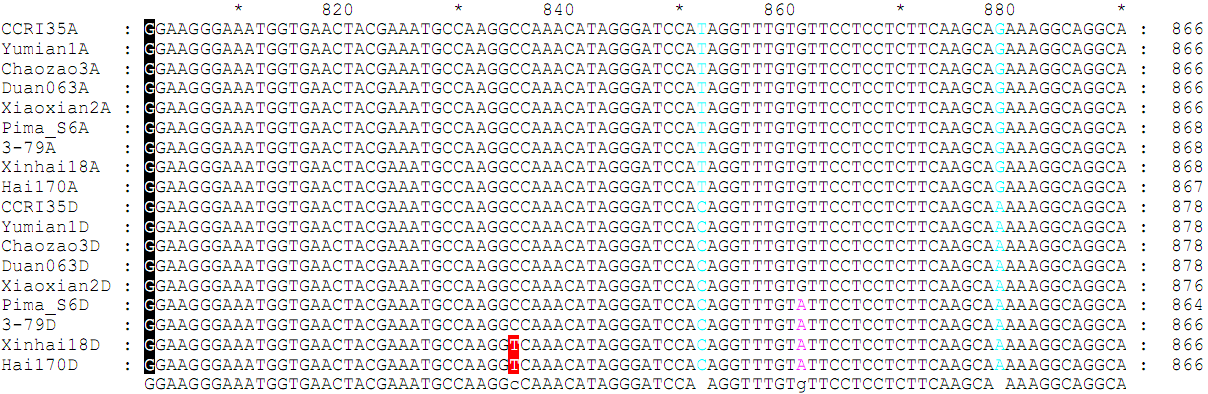


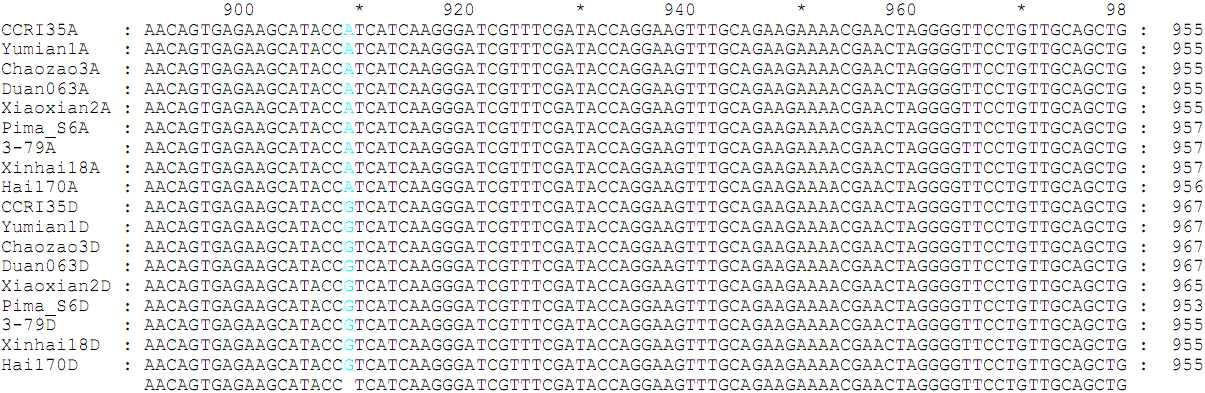


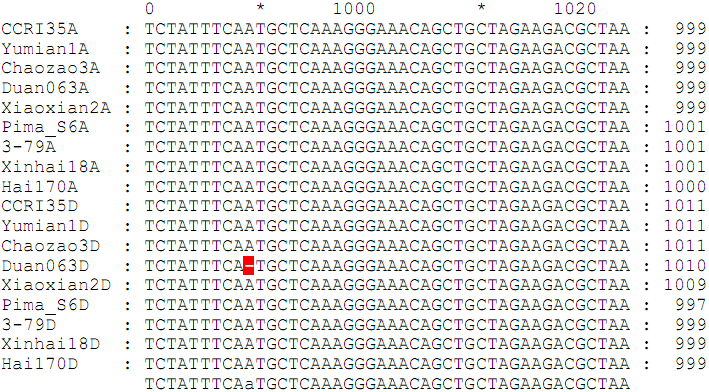


**Fig. S5.** **The nucleotide sequence alignment of *GoCEN* genesfrom A-subgenome and D-subgenome from cotton cultivars/lines used in this study.** Exons are in white background; Introns are inblack background. Nucleobases in blue-green showed the difference between homoeologous *CEN-At* and *CEN-Dt*. Nucleobases in violet showed the difference of *GoCEN* in the same subgenome between *G. barbadense* and *G. hirsutum*. Nucleobases in the red background show the *cl* mutations: “C” nucleotide replaced by “T” nucleotide in 825 bp in Hai170 and Xinhai18; “G” nucleotide replaced by “A” nucleotide in 304 bp in Hai170 and Xinhai18; “A” nucleotide deletion between 898 bp and 900 bp in Duan063; “CA” nucleotides deletion between 350 bp and 353 bp in Xiaoxian2.


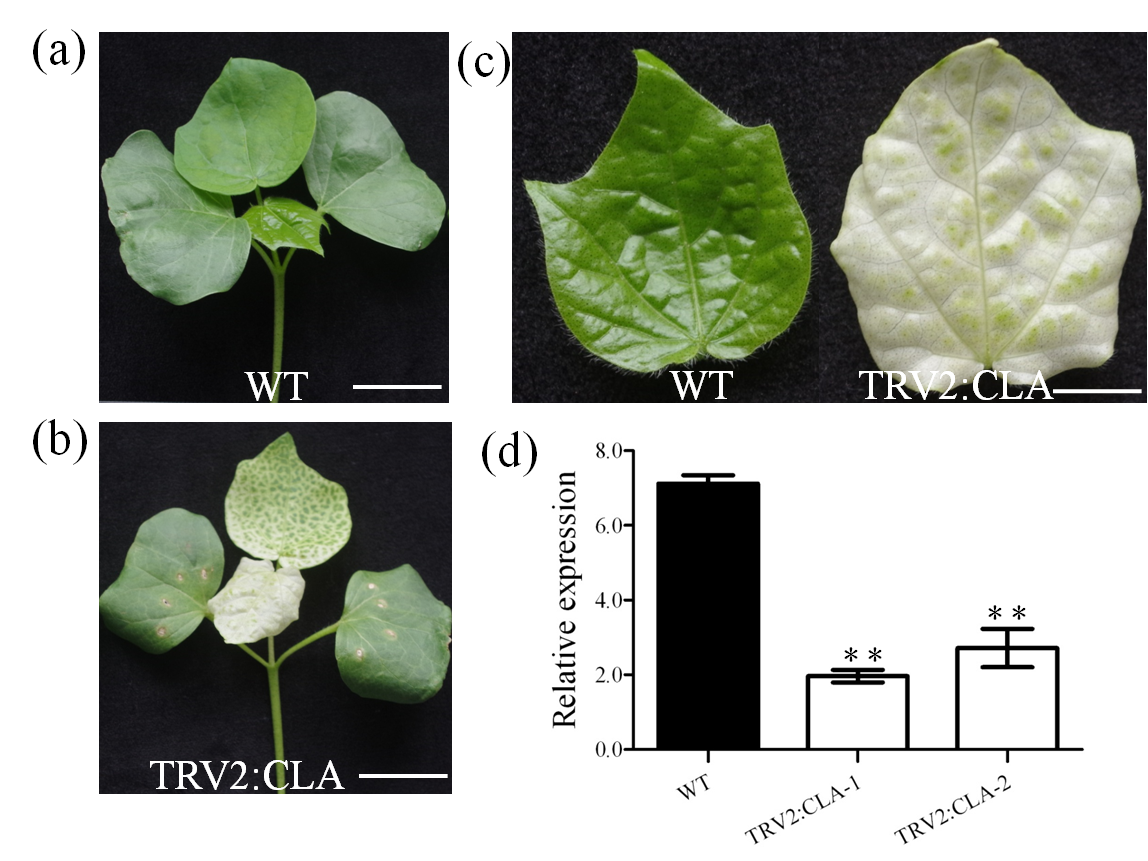


**Fig. S6. Functional characterization of Go*CLA* by VIGS.**

(a) (b) Plant morphologies of WT and plant Go*CLA* silencing by VIGS after twenty days post inoculation; Scale bars, 2cm. (c) Showing the presence and the absence of chloroplast in WT and plant Go*CLA* silencing by VIGS; Scale bars, 0.5cm . (d) Transcript level of Go*CLA* in normal and corresponding Go*CLA* -silenced leaves. The error bar represents the standard deviation of the mean values of three biological replicates. ** = p<0.01, student’s t-test, n=3. The actin gene was used as the internal control.


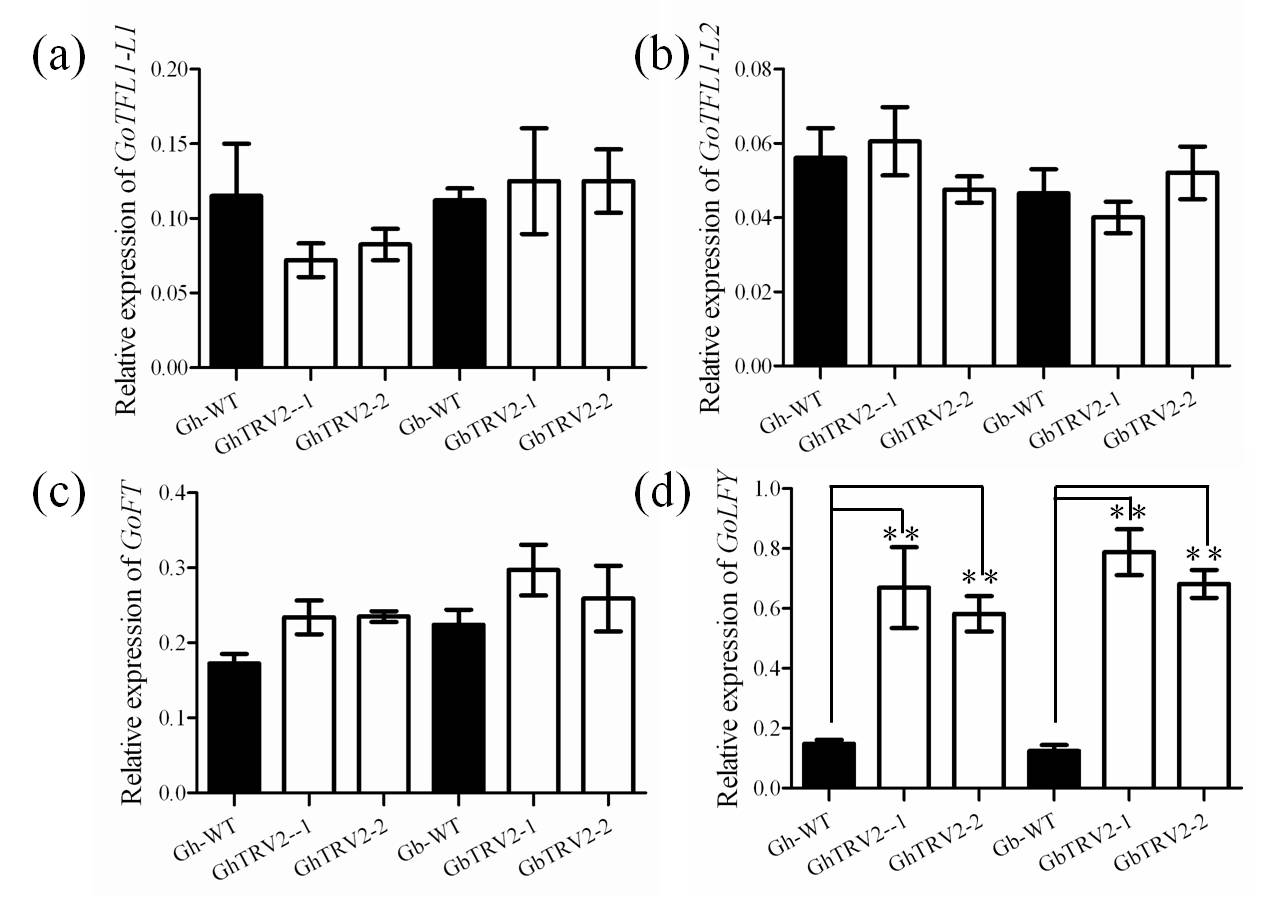


**Fig. S7. Genes expression level with RT-PCR in *G. hirsutum* and *G. barbadense* between CEN-silenced and WT plant.** TFL1-L1 and TFL1-L2 share homology with CEN (AtTFL1) gene in cotton. The error bar represents the standard deviation of the mean values of three biological replicates. ** = p<0.01, student’s t-test, n=3. The actin gene was used as the internal control.


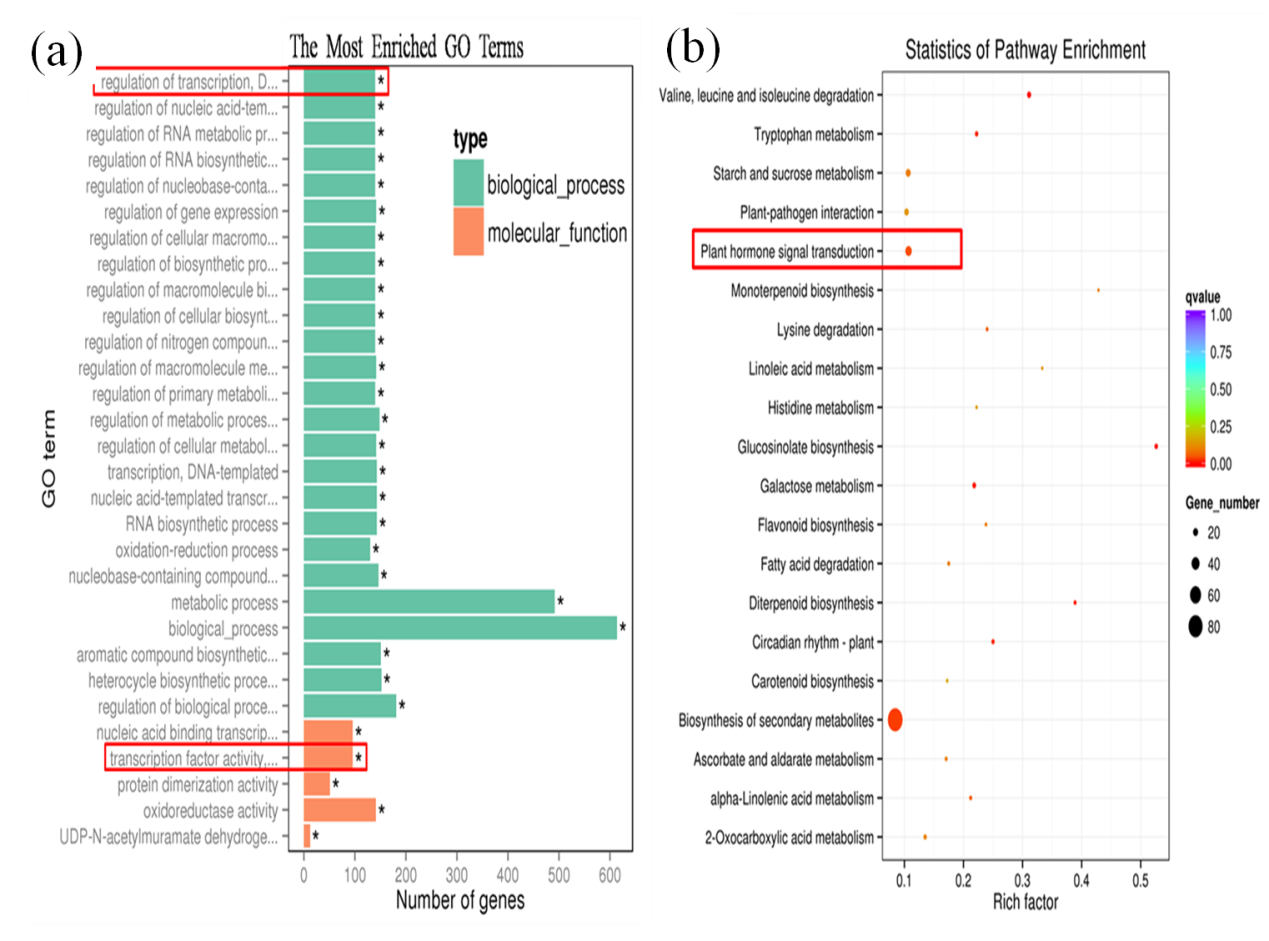


**Fig. S8.** **GO and KEGG enrichment analysis of differentially expressed genes screening from WT and *GoCEN* silenced plant in *G. hirsutum* and *G. barbadense*.** (a) GO enrichment analysis showed that transcription factor activity was enriched in biological process and molecular functional categories. (b) KEGG analysis of DEGS showed that functional category plant hormone signal transduction was enriched.


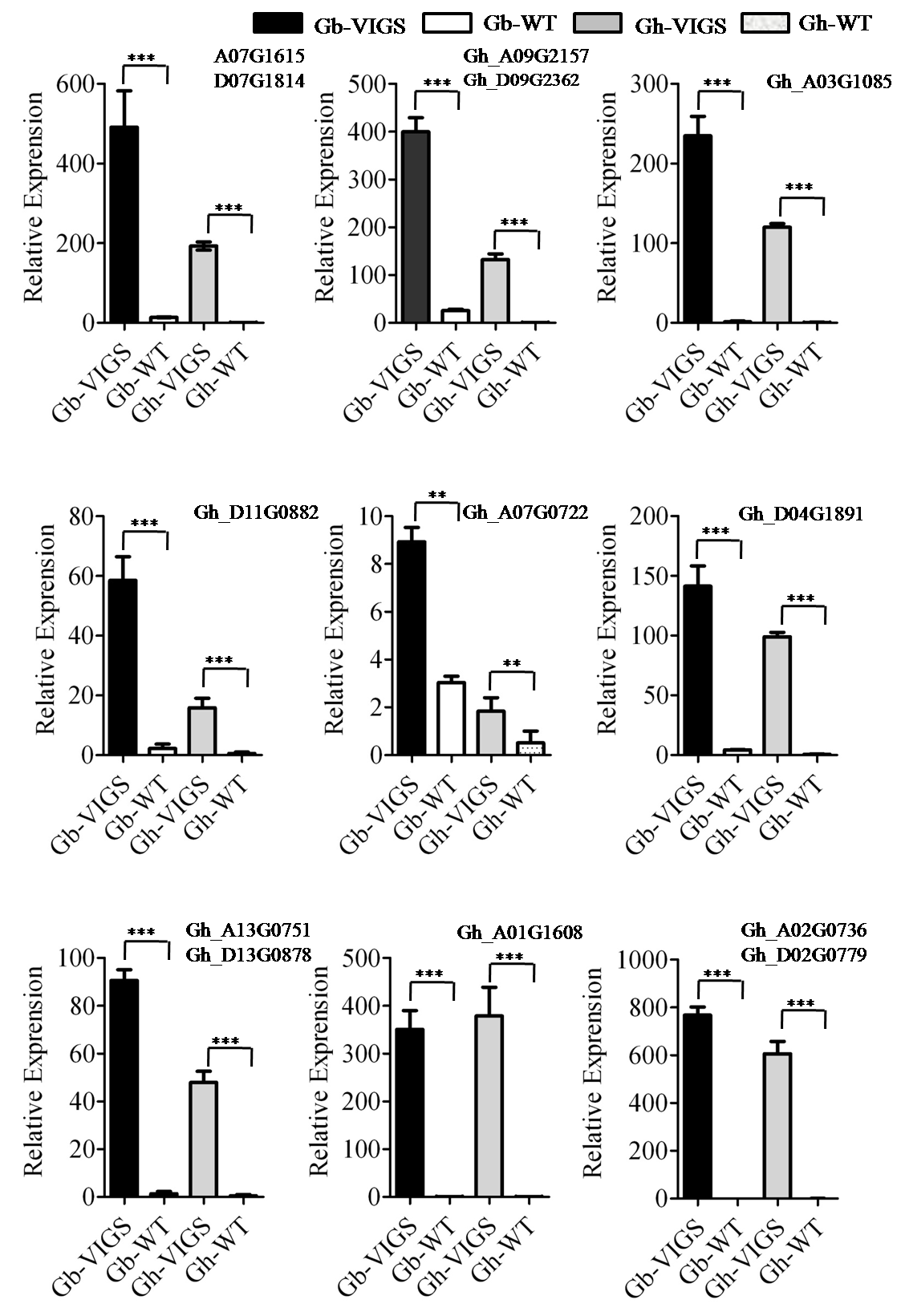


**Fig. S9. qRT-PCR validation of MADS-box transcription factors in *G. hirsutum* and *G. barbadense* between CEN-silenced and WT plant.** The error bar represents the standard deviation of the mean values of three biological replicates. ** = p<0.01, student’s t-test, n=3. The actin gene was used as the internal control.


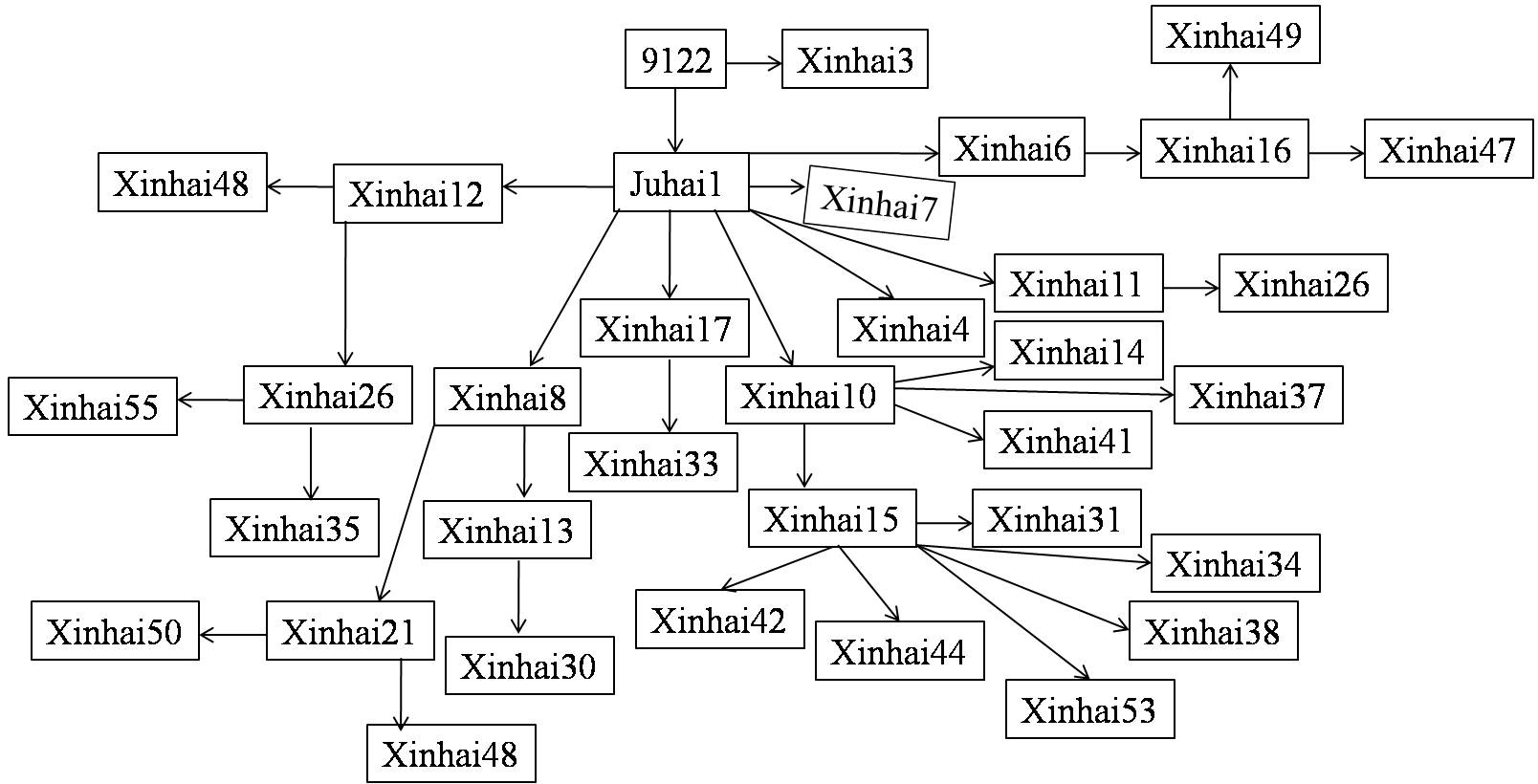


**Fig. S10. The pedigree of *G. barbadense* commercial cultivars in Xinjiang Province.** The arrow represented transfer pathway of hereditary information.

**Table S1 Thirty-six candidate genes for *Gh-cl* and their putative function.**

| Gene ID | Start point | End point | Best-hit-arabi-name | Putative function |
| --- | --- | --- | --- | --- |
| Gohir.D07G111800 | 15638402 | 15639714 | AT2G38640.1 | arabi-symbol |
| Gohir.D07G111900 | 15649281 | 15651240 | AT2G27740.1 |  |
| Gohir.D07G112000 | 15663408 | 15664478 | AT3G22430.1 |  |
| Gohir.D07G112100 | 15674226 | 15676507 | AT1G50300.1 | TAF15 |
| Gohir.D07G112200 | 15701834 | 15706056 | AT3G44610.1 |  |
| Gohir.D07G112300 | 15717796 | 15723971 | AT3G44600.1 | CYP71 |
| Gohir.D07G112400 | 15724276 | 15725693 | AT3G44590.1 |  |
| Gohir.D07G112500 | 15726304 | 15730965 | AT4G04190.1 |  |
| Gohir.D07G112600 | 15733127 | 15736226 | AT4G13650.1 |  |
| Gohir.D07G112700 | 15745224 | 15748743 | AT2G16650.1 | PRORP2 |
| Gohir.D07G112800 | 15747998 | 15753494 | AT4G33790.1 | CER4,FAR3,G7 |
| Gohir.D07G112900 | 15785955 | 15788838 | AT5G53490.3 |  |
| Gohir.D07G113000 | 15813857 | 15816498 | AT2G27610.1 |  |
| Gohir.D07G113100 | 15818271 | 15823297 | AT2G27600.1 | ATSKD1,SKD1,VPS4 |
| Gohir.D07G113200 | 15836587 | 15837992 | AT2G27580.1 |  |
| Gohir.D07G113300 | 15843821 | 15846190 | AT1G08280.1 |  |
| Gohir.D07G113400 | 15863321 | 15866310 | AT1G08290.1 | WIP3 |
| Gohir.D07G113500 | 16047144 | 16048462 | AT2G27550.1 | ATC |
| Gohir.D07G113600 | 16122405 | 16125927 | AT5G22460.2 |  |
| Gohir.D07G113700 | 16180624 | 16186682 | AT1G02205.2 | CER1 |
| Gohir.D07G113800 | 16246216 | 16251477 | AT1G08320.3 | bZIP21,TGA9 |
| Gohir.D07G113900 | 16264618 | 16268915 | AT1G67190.1 |  |
| Gohir.D07G114000 | 16314082 | 16316982 | AT2G27500.1 |  |
| Gohir.D07G114100 | 16317481 | 16321243 | AT1G02410.1 |  |
| Gohir.D07G114200 | 16323397 | 16326133 | AT5G22400.1 |  |
| Gohir.D07G114300 | 16328107 | 16329253 | AT5G22390.1 |  |
| Gohir.D07G114400 | 16355623 | 16356815 | AT3G44380.1 |  |
| Gohir.D07G114500 | 16377853 | 16379250 | AT3G44350.2 | anac061,NAC061 |
| Gohir.D07G114600 | 16379386 | 16379592 |  |  |
| Gohir.D07G114700 | 16397670 | 16401637 | AT5G22350.1 | ELM1 |
| Gohir.D07G114800 | 16403336 | 16407373 | AT2G27350.6 |  |
| Gohir.D07G114900 | 16408735 | 16410180 | AT2G27310.1 |  |
| Gohir.D07G115000 | 16413117 | 16414918 | AT1G08380.1 | PSAO |
| Gohir.D07G115100 | 16434449 | 16437570 | AT3G11590.1 |  |
| Gohir.D07G115200 | 16438158 | 16441354 | AT5G22300.1 | AtNIT4,NIT4 |
| Gohir.D07G115300 | 16451150 | 16451482 | AT3G11600.1 |  |

**Table S2 Nucleotide sequence variations between *GoCEN-A07* and *GoCEN-D07* in tetraploid cotton.**

| Loci (bp) | Base sequence | Exon/Introne | Difference | | |
| --- | --- | --- | --- | --- | --- |
|  |  |  | At/Dt | At of *G. hirsutum* | Dt of *G. barbadense* |
| 28 | G/T | Exon | √ | — | — |
| 217 | A/G | Introne | — | √ | — |
| 229 | C/T | Introne | √ | — | — |
| 247 | T/G | Introne | √ | — | — |
| 248-250 | TCT/G | Introne | √ | — | — |
| 252-253 | GA/AT | Introne | √ | — | — |
| 397 | A/C | Introne | √ | — | — |
| 401 | T/- | Introne | √ | — | — |
| 412 | C/T | Introne | √ | — | — |
| 468-469 | --/CT | Introne | √ | — | — |
| 472 | C/A | Introne | √ | — | — |
| 484 | C/T | Introne | — | — | √ |
| 521-523 | AAA/--- | Introne | √ | — | — |
| 687 | T | Introne | — | — | √ |
| 706-727 | SSR | Introne | √ | √ | √ |
| 781 | T/A | Introne | √ | — | — |
| 790 | T/- | Introne | √ | — | — |
| 791-792 | AT/-- | Introne | √ | — | — |
| 794-796 | ACA/--- | Introne | √ | — | — |
| 852 | T/C | Exon | √ | — | — |
| 861 | A/G | Exon | — | — | √ |
| 880 | C/A | Exon | √ | — | — |
| 909 | A/G | Exon | √ | — | — |

**Table S3 MADS-box transcription factors differentially expressed in transcriptome**

| Gene_ID in cotton | **Gene symbol** | ***Arabidopsis* ID** | FPKM in Gh_VIGS | FPKM in Gh_WT | log2FoldChange | FPKM in Gb_VIGS | FPKM in Gb_WT | log2FoldChange |
| --- | --- | --- | --- | --- | --- | --- | --- | --- |
| *Gh_A04G1264* | AP1 | AT1G69120 | 138.8 | 0.9 | 7.3 | 299.7 | 6.4 | 5.6 |
| *Gh_A13G0751* |  |  | 265.9 | 2.3 | 6.9 | 518.0 | 9.7 | 5.7 |
| *Gh_D02G1311* |  |  | 78.2 | 26.0 | 1.6 | 298.9 | 20.6 | 3.9 |
| *Gh_D04G1891* |  |  | 188.3 | 0.8 | 7.8 | 481.3 | 12.8 | 5.2 |
| *Gh_D13G0878* |  |  | 232.2 | 3.1 | 6.2 | 338.8 | 11.3 | 4.9 |
| *Gh_A07G1339* | AGL6 | AT2G45650 | 26.6 | 0.4 | 6.0 | 47.1 | 0.5 | 6.6 |
| *Gh_A08G1148* |  |  | 20.7 | 0.0 | Inf | 16.1 | 0.0 | Inf |
| *Gh_A11G0754* |  |  | 43.8 | 0.5 | 6.5 | 53.3 | 4.4 | 3.6 |
| *Gh_D08G1430* |  |  | 40.4 | 0.0 | Inf | 116.9 | 3.0 | 5.3 |
| *Gh_D09G0390* |  |  | 463.4 | 192.5 | 1.3 | 181.3 | 42.2 | 2.1 |
| *Gh_D11G0882* |  |  | 75.0 | 4.6 | 4.0 | 101.2 | 2.9 | 5.1 |
| *Gh_A10G2220* | AG | AT4G18960 | 117.1 | 38.0 | 1.6 | 40.8 | 9.7 | 2.1 |
| *Gh_D10G0308* |  |  | 19.6 | 0.0 | Inf | 28.8 | 0.5 | 5.8 |
| *Gh_D10G0309* |  |  | 36.4 | 0.4 | 6.4 | 45.1 | 0.0 | Inf |
| *Gh_A07G1615* | SEP2 | AT3G02310 | 102.3 | 2.3 | 5.5 | 105.8 | 6.9 | 3.9 |
| *Gh_A09G2157* |  |  | 36.1 | 0.9 | 5.3 | 29.2 | 0.5 | 5.9 |
| *Gh_D07G1814* |  |  | 58.2 | 0.4 | 7.1 | 50.0 | 1.4 | 5.1 |
| *Gh_D09G2362* |  |  | 64.9 | 0.0 | Inf | 49.6 | 5.8 | 3.1 |
| *Gh_A03G1085* | SEP3 | AT1G24260 | 103.4 | 1.4 | 6.2 | 59.3 | 1.0 | 5.9 |
| *Gh_D02G1502* |  |  | 64.9 | 0.0 | Inf | 48.4 | 0.5 | 6.7 |
| *Gh_A04G1265* | SEP4 | AT2G03710 | 170.0 | 69.2 | 1.3 | 407.2 | 39.6 | 3.4 |
| *Gh_D13G0877* |  |  | 166.1 | 23.2 | 2.8 | 162.0 | 2.4 | 6.1 |
| *Gh_A02G1617* | AP3 | AT3G54340 | 35.1 | 0.4 | 6.4 | 31.7 | 1.9 | 4.1 |
| *Gh_A12G0570* |  |  | 28.7 | 6.7 | 2.1 | 46.3 | 1.0 | 5.5 |
| *Gh_D03G0105* |  |  | 25.7 | 1.0 | 4.7 | 53.8 | 16.1 | 1.7 |
| *Gh_D05G2452* |  |  | 91.8 | 11.8 | 3.0 | 41.5 | 14.1 | 1.6 |
| *Gh_A01G1608* | PI | AT5G20240 | 101.9 | 0.0 | Inf | 111.4 | 0.0 | Inf |
| *Gh_A02G0736* |  |  | 91.7 | 0.0 | Inf | 79.1 | 0.0 | Inf |
| *Gh_D02G0779* |  |  | 85.1 | 0.0 | Inf | 81.4 | 0.0 | Inf |
| *Gh_Sca007246G01* |  |  | 85.1 | 0.0 | Inf | 65.2 | 0.5 | 7.1 |
| *Gh_A05G1797* | AGL104 | AT1G22130 | 565.2 | 131.4 | 2.1 | 116.5 | 14.8 | 3.0 |
| *Gh_D05G1992* |  |  | 654.3 | 112.3 | 2.5 | 108.6 | 12.8 | 3.1 |
| *Gh_D07G0780* | AGL8 | AT5G60910 | 133.2 | 61.0 | 1.1 | 236.0 | 97.1 | 1.3 |
| *Gh_D12G2226* | AGL19 | AT4G22950 | 18.2 | 104.6 | -2.5 | 2.0 | 15.7 | -3.0 |
| *Gh_A07G0722* | CAL | AT1G26310 | 185.4 | 39.2 | 2.2 | 108.4 | 18.2 | 2.6 |
| *Gh_A13G0442* | SVP | AT2G22540 | 79.5 | 9.2 | 3.1 | 8.6 | 29.8 | -1.8 |
| *Gh_D11G0400* | SPH2 | AT2G42830 | 174.8 | 82.7 | 1.1 | 200.6 | 18.0 | 3.5 |

Inf indicates that the gene is undetectable in wild type plant and its expression level is significantly up-regulated in *CEN*-silenced cotton.
